# Prevalence of malnutrition and nutritional risk, and associated factors in adults with head and neck cancer: a systematic review and meta-analysis

**DOI:** 10.3389/fonc.2026.1905663

**Published:** 2026-08-12

**Authors:** Qianqian Li, Yin Chen, Chunxiang Chen, Meizhen Ye

**Affiliations:** 1 Clinical Oncology School of Fujian Medical University, Fujian Cancer Hospital, Fuzhou, Fujian, China; 2 The Second Affiliated Hospital of Xiamen Medical College, Xiamen, Fujian, China

**Keywords:** associated factors, head and neck cancer, malnutrition, meta-analysis, nutritional screening, systematic review

## Abstract

**Background:**

Head and neck cancer (HNC) carries a high burden of malnutrition due to tumor location, treatment toxicity, and systemic inflammation. However, prevalence estimates vary across assessment instruments, and associated clinical factors have not been comprehensively quantified. This systematic review and meta-analysis estimated the prevalence of diagnostically defined malnutrition, nutritional risk, and PG-SGA-based nutritional categories and summarized factors associated with these outcomes in adults with HNC.

**Methods:**

A systematic literature search was conducted in the databases PubMed, Embase, Web of Science, Cochrane Library, CINAHL, CNKI, Wanfang, VIP, and CBM databases from inception to July, 2026. Research reports on malnutrition, nutritional risk and related factors for unhealthy weight in adults were included. Joanna Briggs Institute (JBI) critical appraisal checklist was employed to assess the methodological quality of prevalence studies, and the Quality in Prognosis Studies (QUIPS) tool was used for associated-factor studies. Pooled prevalence estimates were calculated using random-effects models and restricted maximum likelihood (REML) estimation in the logit scale. Generalized Linear Mixed Models and alternative transformations were used for sensitivity analysis.

**Results:**

Twenty studies involving 4,456 participants were included. The pooled prevalence of GLIM-defined malnutrition was 39.4% (95% CI: 30.4–49.1%; five studies, 1,158 participants), PG-SGA-based nutritional categories 57.8% (95% CI: 37.7–75.7%; six studies, 724 participants), and NRS 2002-defined nutritional risk 32.0% (95% CI: 18.9–48.7%; six studies, 1,423 participants). Considerable heterogeneity was observed across all assessment-specific analyses. Frequently reported associated factors included advanced age, lower body mass index, reduced serum albumin or prealbumin, treatment-related burden, radiotherapy exposure, and disease characteristics. Most studies had low-to-moderate or moderate methodological limitations, primarily related to observational designs, residual confounding, and incomplete reporting of analytical methods.

**Conclusion:**

Malnutrition and nutritional risk are common in patients with HNC, although estimates depend on the outcome definition and assessment instrument. GLIM provides a diagnostic definition of malnutrition, PG-SGA classifies nutritional status, and NRS 2002 identifies nutritional risk rather than confirmed malnutrition. Because the evidence was predominantly observational and geographically heterogeneous, the findings should be interpreted as descriptive and associative rather than causal. Risk-stratified nutritional screening and multidisciplinary assessment should therefore be considered in clinical practice.

**Systematic review registration:**

https://www.crd.york.ac.uk/PROSPERO/, identifier CRD420261418334.

## 1 Introduction

Head and neck cancer (HNC) encompasses a diverse group of malignancies arising from the mucosal surfaces of the upper aerodigestive tract, including the oral cavity, pharynx, larynx, paranasal sinuses, and salivary glands. HNC is the seventh most common cancer worldwide, and about 900,000 new cases and 450,000 deaths occur every year (1). The disease burden is relatively high in the developing areas of South and Southeast Asia, and tobacco use, betel nut chewing and widespread human papillomavirus infection all contribute to elevated prevalence rates (2). Squamous cell carcinoma constitutes more than 90% of all HNC cases, with nasopharyngeal carcinoma (NPC) demonstrating distinct epidemiological patterns concentrated in Southern China and Southeast Asia (3).

Malnutrition in patients with HNC is a pervasive and clinically significant problem caused by a combination of factors such as tumor-related, treatment-related, and patient-specific factors (4). The anatomical location of HNC tumors directly compromises oral intake through mechanical obstruction, pain, dysphagia, and alterations in taste and smell perception (5). Furthermore, the catabolic effects of malignancy induce systemic inflammatory responses that accelerate protein catabolism and deplete energy reserves (6). Oncologic treatments, particularly radiotherapy and chemoradiotherapy, frequently exacerbate nutritional deterioration by causing mucositis, xerostomia, odynophagia, and nausea, thereby creating a vicious cycle of declining oral intake and progressive nutritional decline (7).

The health problems of malnutrition in HNC are far-reaching and well-documented. Malnourished patients exhibit impaired wound healing, increased susceptibility to infectious complications, reduced tolerance to aggressive oncologic therapies, and experience prolonged hospital stays (8, 9). Population-based studies have consistently demonstrated that pre-treatment malnutrition is an independent prognostic factor for decreased overall survival and disease-free survival in HNC (10, 11). In addition, nutritional compromise adversely affects patients’ quality of life, functional status, and psychological well-being; therefore, it will be more difficult for them to cope with cancer (12).

Although nutritional status is known to be related to the course of HNC, the reported prevalence of malnutrition varies considerably across published studies, ranging from approximately 15% to over 80% (9). This variation reflects differences in patient populations, cancer subsites, treatment modalities, and nutritional assessment methods. Several validated instruments are used in clinical practice, including the Global Leadership Initiative on Malnutrition (GLIM) criteria, the Patient-Generated Subjective Global Assessment (PG-SGA), and Nutritional Risk Screening 2002 (NRS 2002) (13, 14). These instruments have different conceptual bases, cut-off thresholds and clinical goals, and are thus inconsistent in the reported prevalence.

A substantial body of observational research has investigated factors associated with malnutrition in HNC, identifying demographic characteristics, lifestyle behaviors, biochemical markers, tumor-related characteristics, functional symptoms, and treatment variables as potential correlates of nutritional status (15, 16). However, individual studies are often limited by small sample sizes, single-center designs, cross-sectional measurements, and inconsistent variable definitions, resulting in conflicting or inconclusive findings. A 2024 systematic review narratively categorized malnutrition-associated factors in HNC but did not quantitatively pool prevalence or comparable association estimates (11). The present review therefore extends the search through July 2026, incorporates Chinese-language databases, distinguishes nutritional outcome constructs, and quantitatively synthesizes tool-specific prevalence and comparable associated-factor estimates.

The objectives of this systematic review and meta-analysis were threefold: first, to estimate the pooled prevalence of diagnostically defined malnutrition, nutritional risk, and PG-SGA–based nutritional categories among patients with HNC, with estimates stratified by nutritional assessment tool and outcome definition; second, to identify and quantify the associations between patient-related, disease-related, functional, biochemical, and treatment-related variables with malnutrition or nutritional risk; and third, to synthesize the available evidence to support the development of clinical nutritional screening and management strategies for this vulnerable patient population.

## 2 Materials and methods

This systematic review and meta-analysis was conducted in accordance with the Preferred Reporting Items for Systematic Reviews and Meta-Analyses (PRISMA) 2020 guidelines (17). The review was registered in PROSPERO as a systematic review registration (CRD420261418334) on 8 June 2026, and the registration record is publicly available at https://www.crd.york.ac.uk/PROSPERO/view/CRD420261418334. A full updated search was subsequently conducted on July 2026 before resubmission. The PROSPERO record states that no separate full protocol document had been prepared; therefore, the registration record was used to document the prespecified review scope and methods. Deviations from the registered record, including the expansion of databases, revision of terminology from “risk factors” to “associated factors,” and the addition of detailed evidence-traceability and reproducibility materials, are summarized in the **Supplementary Materials**.

### 2.1 Inclusion and exclusion criteria

Inclusion criteria were defined using a PECO-informed prognostic-factor framework, because the review focused on variables associated with malnutrition or nutritional rather than on intervention effects requiring a formal comparator. The eligibility criteria were as follows: (1) Population: adult patients (≥18 years) diagnosed with head and neck cancer, including cancers of the oral cavity, oropharynx, nasopharynx, hypopharynx, larynx, and other sites within the head and neck region; (2) Exposure or associated variable: demographic, behavioral, biochemical, functional, tumor-related, treatment-related, or psychosocial variables examined in relation to malnutrition, nutritional risk, or PG-SGA-based nutritional category; (3) Comparator or reference category: the non-exposed or lower-risk category reported in each original study, such as younger age, normal BMI, non-smoking status, normal albumin or hemoglobin level, absence of dysphagia, early tumor stage, or absence of radiotherapy/chemoradiotherapy; (4) Outcome: outcomes were separated into GLIM-defined or otherwise diagnostically defined malnutrition, NRS 2002-defined nutritional risk, PG-SGA-based nutritional categories, and prospective prevalence only when new cases were observed among patients free of the specified outcome at baseline; and (5) Study design: observational studies, including cross-sectional studies, cohort studies, case-control studies, longitudinal studies, and secondary analyses of randomized controlled trials reporting baseline or follow-up nutritional data.

**Exclusion criteria** comprised: (1) studies reporting exclusively on pediatric populations; (2) case reports, case series with fewer than 10 participants, narrative reviews, editorials, and conference abstracts without sufficient methodological detail; (3) studies in which malnutrition was not the primary outcome or in which associated-factor data were not extractable; (4) duplicate publications or overlapping cohorts, in which case the most comprehensive or recent publication was retained; and (5) studies focusing exclusively on cancer cachexia syndrome without broader malnutrition assessment.

### 2.2 Search strategy

A comprehensive literature search was conducted using the 6S evidence pyramid to improve retrieval sensitivity and reduce the risk of missing relevant evidence (18). Sources were searched hierarchically, beginning with synthesized evidence resources and proceeding to primary-study databases.

Synthesized evidence sources and guideline repositories at the upper levels of the 6S pyramid were searched first, with emphasis on head and neck cancer, oncology care, and clinical nutrition. These sources included UpToDate, BMJ Best Practice, the World Health Organization (WHO) Global Cancer Observatory and related WHO resources, Joanna Briggs Institute (JBI) Evidence Synthesis, and guidelines issued by the American Society of Clinical Oncology (ASCO), European Society for Medical Oncology (ESMO), European Society for Clinical Nutrition and Metabolism (ESPEN), American Society for Parenteral and Enteral Nutrition (ASPEN), and other relevant head-and-neck oncology and nutrition organizations.

A comprehensive literature search was conducted in PubMed/MEDLINE via NCBI, Embase via Elsevier, Web of Science Core Collection via Clarivate, Cochrane Library via Wiley, CINAHL via EBSCOhost, China National Knowledge Infrastructure (CNKI), Wanfang Data, VIP Database, and Chinese Biomedical Literature Database (CBM/SinoMed). The initial search covered database inception to 31 May 2025, and a full updated search was performed on 4 July 2026 to capture studies published before manuscript resubmission. No date restriction was applied at the search stage. English and Chinese language restrictions were applied only during eligibility assessment. The search strategy was developed by two reviewers and checked against the PRESS 2015 recommendations for electronic search strategies (19).

The search combined controlled vocabulary and free-text terms for three core concepts: head and neck cancer, malnutrition or nutritional status, and associated factors or prevalence. Search terms were adapted for each database according to its indexing system and syntax. The PubMed strategy combined MeSH terms and title/abstract terms; Embase combined Emtree terms and title/abstract terms; Web of Science used topic fields; Cochrane Library used title/abstract/keyword fields; CINAHL used CINAHL Headings and free-text fields; and Chinese databases used Chinese subject terms and free-text terms. The complete database-specific strategies, platforms, search dates, and record counts are reported in **Supplementary Table 2**. Reference lists of all included studies and relevant systematic reviews were hand-searched. Additional records were also identified from oncology and nutrition guideline repositories, including ESPEN, ASPEN, ASCO, ESMO, and head-and-neck oncology guideline sources. All retrieved citations were exported in RIS or compatible formats and imported into EndNote X9 for deduplication before screening.

### 2.3 Literature screening and data extraction

All records were exported from each database in RIS or compatible formats and imported into EndNote X9 (Clarivate Analytics). Deduplication was conducted in three sequential steps. First, EndNote’s automatic duplicate function was applied using DOI, PMID, title, author, year, journal, volume, and page fields. Second, two reviewers manually checked potential duplicates with minor title variation, abbreviated journal names, translated Chinese-English titles, and missing DOI/PMID information. Third, overlapping cohort publications were reviewed at the full-text stage; when multiple publications appeared to report the same or substantially overlapping cohort, the most complete, recent, or methodologically informative report was retained. After removal of 1,712 duplicate records, 4,727 unique records remained for title and abstract screening. Title and abstract screening and subsequent full-text eligibility assessment were conducted independently by two reviewers using prespecified inclusion and exclusion criteria. The original title and abstract screening was conducted from 1 June to 20 July 2025, followed by full-text eligibility assessment from 21 July to 30 September 2025. Initial data extraction was completed between 1 October and 15 December 2025. Following the updated search on 4 July 2026, newly identified records were screened from 5 to 10 July 2026, and the updated extraction, reference verification, and cohort-independence audit were completed from 11 to 15 July 2026.

The screening decision categories, eligibility criteria applied at the full-text stage, and corresponding decision rules are summarized in **Supplementary Table 4**. For each report assessed in full text, the final eligibility decision and the principal reason for exclusion were recorded using mutually exclusive categories. The full-text exclusion categories and their definitions are provided in **Supplementary Table 5**. Disagreements were resolved through discussion and, when required, adjudication by a third reviewer. A study-level extraction table was additionally prepared to document the effect estimate used for each associated-factor analysis, including the effect-measure type, adjustment status, covariates included in the original model, reference category, variable cut-off, exposure timing, outcome timing, and whether the estimate entered the primary meta-analysis or was retained for sensitivity or descriptive analysis.

A standardized data extraction form was developed *a priori* and piloted on five randomly selected studies. Two reviewers independently extracted the following data elements: (1) study characteristics (first author, publication year, country, study design, sample size, cancer site, assessment period); (2) participant demographics (mean age, proportion male, proportion smokers); (3) nutritional assessment methodology, including the assessment instrument, exact cut-off, case definition, assessment time point, event count, and whether the outcome represented diagnostically defined malnutrition, nutritional risk, PG-SGA-based nutritional category, or prospective prevalence; (4) associated-factor data, including the measurement method, cut-off value, exposure or assessment timing, outcome assessment timing, follow-up period where applicable, reference category, effect-measure type, adjustment status, adjusted covariates, and unadjusted or adjusted ORs, RRs, or HRs with 95% CIs, or raw frequencies enabling calculation of effect estimates; and (5) funding source and conflict of interest declarations.

When studies reported multiple nutritional assessment tools, each tool-specific outcome was extracted as a separate data point and assigned to the appropriate outcome construct. GLIM-based outcomes were treated as diagnostically defined malnutrition; NRS 2002 outcomes were treated as nutritional risk; PG-SGA outcomes were reported according to the nutritional category or cut-off used in the original study; and prospective prevalence was extracted only if the original study documented new cases during follow-up among participants without the specified nutritional outcome at baseline. **Table 1** summarizes abbreviated definitions and **Supplementary Table 1** provides detailed study-specific cut-offs or operational definitions where available. For associated-factor analyses, adjusted odds ratios were preferentially extracted when available; otherwise, unadjusted estimates were calculated from raw contingency tables. Authors were contacted by email when critical data elements were missing or ambiguous. Before quantitative synthesis, a study-level evidence audit was undertaken by cross-checking each record across **Table 1**, the forest plots, the reference list, and the analytic dataset. The audit fields comprised the full citation and DOI/PMID or database identifier, recruitment setting and period, population, sample size, design, nutritional instrument and cut-off, assessment time point, event count, effect-measure type, adjustment status, covariates, and the exact synthesis to which the record contributed. Generic study labels were replaced by the verified first author and publication year. Records with an unresolved bibliographic identity, an outcome definition incompatible with the prespecified construct, or an untraceable estimate were not considered eligible for a definitive pooled analysis.

**Table 1:** Basic characteristics and quality assessment of included studies.

ID	Author	Country	Recruitment setting and period	Study design	Cancer site	Event count/total	Malnutrition rate (%)	Tool/case definition	Adjustment/covariates	Included meta-analysis	Quality score	Quality level
1	Hu et al. (2025) (15)	China	Zhejiang Cancer Hospital; perioperative HNC cohort; follow-up to 2 months postoperatively	Observational longitudinal	HNC overall	38/148	25.7	NRS 2002 ≥3	Adjusted for demographic, clinical, biochemical, and treatment-related variables reported in the original logistic model	NRS 2002 prevalence; advanced age; low albumin; low hemoglobin; sensitivity analysis	8	High
2	Steer et al. (2020) (16)	Australia	Cancer treatment services; inpatient and ambulatory HNC patients	Cross-sectional	HNC overall	61/217	28.1	≥1 phenotypic + ≥1 etiologic GLIM criterion	Mainly unadjusted prevalence data; clinical variables extracted when available	GLIM prevalence; treatment-related subgroup evidence	7	Moderate
3	Einarsson et al. (2020) (29)	Sweden	Hospital-based HNC treatment cohort	Observational	HNC overall	18/57	31.6	GLIM phenotypic + etiologic criteria	Adjustment not uniformly available; clinical and treatment variables recorded	GLIM prevalence; advanced age; treatment-phase evidence	7	Moderate
4	Orell et al. (2022) (30)	Finland	Ambulatory patients with newly diagnosed HNC	Prospective cohort	HNC overall	24/65	36.9	GLIM phenotypic + etiologic criteria	Clinical variables and treatment-related variables considered where reported	GLIM prevalence; smoking; treatment-related evidence	8	High
5	Wang et al. (2023) (31)	China	First Affiliated Hospital of Guangxi Medical University; Sep 2021–Nov 2022	Cross-sectional	NPC	143/305	46.9	≥1 phenotypic + ≥1 etiologic GLIM criterion	Adjusted for BMI, radiation dose, appetite loss, prealbumin and other clinical variables reported in multivariable analysis	GLIM prevalence; low BMI; low albumin; treatment dose; associated-factor analysis	8	High
6	Zhan et al. (2025) (32)	China	NPC patients undergoing radiotherapy; hospital-based cohort	Retrospective	NPC	118/158	74.7	Moderate/severe malnutrition per source PG-SGA definition	Adjusted for clinical and treatment-related predictors selected in the predictive model	PG-SGA prevalence; advanced stage; radiotherapy-related variables	7	Moderate
7	Taouchikht et al. (2024) (33)	Morocco	National Institute of Oncology, Rabat; Oct 2023–Mar 2024	Prospective observational	HNC receiving RT	24/31	77.4	PG-SGA B/C	Mainly unadjusted longitudinal nutritional-status comparison	PG-SGA prevalence; radiotherapy-related nutritional deterioration	6	Moderate
8	Lima et al. (2026) (34)	Brazil	Tertiary reference outpatient clinic; baseline Jul 2017–Nov 2018; follow-up to Nov 2023	Prospective cohort	HNSCC	52/84	61.9	PG-SGA B/C	Ordinal logistic regression and diagnostic-performance analysis; covariates reported in original study	PG-SGA prevalence; diagnostic assessment evidence	9	High
9	Noronha et al. (2025) (35)	India	Tata Memorial Hospital; phase III trial secondary analysis; 2013–2017	Secondary RCT analysis	Laryngeal/hypopharyngeal SCC	64/112	57.1	PG-SGA ≥4	Compared treatment arms, sex, tobacco use, tumor site and survival outcomes; no uniform multivariable malnutrition-risk model	PG-SGA prevalence; chemoradiotherapy-related nutritional deterioration	8	High
10	Deng et al. (2023) (36)	China	Hospital-based HNC radiotherapy cohort	Cross-sectional	HNC receiving RT	52/108	48.1	NRS 2002 ≥3	Sociodemographic, laboratory, clinical and radiotherapy-related variables considered	NRS 2002 prevalence; low albumin; low hemoglobin; radiotherapy-related factors	7	Moderate
11	Wang et al. (2024) (37)	China	NPC malnutrition nomogram development cohort	Model-development study	NPC	116/383	30.3	Definition reported in source model	Adjusted for age, chemotherapy cycles, radiation dose, BMI, albumin, chloride and other model variables	NPC malnutrition model; advanced age; low BMI; low albumin; treatment exposure	7	Moderate
12	Zhu et al. (2025) (38)	China	Nanfang and Zhujiang Hospitals; Jan 2021–Jan 2023; after RT	Two-center retrospective	NPC after RT	116/383	30.3	NRS 2002 threshold reported in source article	Multivariable logistic regression; BMI, chemotherapy cycles, central venous catheter, ALT and other clinical indicators included	NRS 2002 prevalence; post-radiotherapy nutritional risk; model-based associated factors	9	High
13	Wang et al. (2023) (39)	China	Perioperative oral-cancer cohort	Longitudinal	Oral cancer	104/198	52.5	NRS 2002 ≥3	Adjusted for perioperative clinical, demographic and nutritional variables where reported	NRS 2002 prevalence; advanced age; low BMI; perioperative nutritional risk	8	High
14	Chen et al. (2023) (40)	China	Oral-cancer cohort followed through early postoperative period	Longitudinal	Oral cancer	46/269	17.1	PG-SGA malnutrition category per source	Adjusted for perioperative clinical variables, tube feeding, albumin and treatment-related variables where reported	PG-SGA prevalence; low albumin; prolonged tube feeding; postoperative radiotherapy	7	Moderate
15	Liu et al. (2024) (41)	China	HNC/NPC radiotherapy cohort in Nanning	Observational	HNC/NPC receiving RT	137/281	48.8	Source-defined PNI trajectory	Adjusted for prognostic nutritional index trajectory, mucositis-related variables and treatment factors	Combined-tool prevalence; radiotherapy-related nutritional deterioration	6	Moderate
16	Tu et al. (2024) (42)	China	Zhejiang Cancer Hospital; Jan 2017–Dec 2020	Observational comparative	Perioperative HNSCC	18/209	8.6	NRS 2002 ≥3	Mainly diagnostic-comparison analysis; perioperative variables considered	NRS 2002 prevalence; tool-comparison evidence	8	High
17	Wang et al. (2024) (43)	China	Guangxi clinical cohort; Jan 2022–May 2023	Prospective cross-sectional	NPC	155/377	41.1	NRS 2002 ≥3	Adjusted for age, BMI, radiation-treatment number, albumin and other clinical variables	NRS 2002 prevalence; advanced age; low BMI; low albumin; radiotherapy exposure	8	High
18	Vílchez-López et al. (2024) (44)	Spain	VALOR Group multicenter cohort; first 2 weeks of RT	Multicenter observational	HNC overall	265/514	51.6	GLIM criteria	Adjusted for clinical, biochemical, morphofunctional and treatment-related variables where reported	GLIM prevalence; biochemical and treatment-related associated factors	9	High
19	Kuang et al. (2025) (45)	China	Tertiary hospital; Aug 2023–May 2024	Prospective model-development	Postoperative oral cancer	251/487	51.5	GLIM-defined postoperative malnutrition	Model variables included age, tumor stage, reconstruction, diabetes, lymphocyte count, cholesterol and other predictors	Postoperative prevalence; advanced stage; age; model-based associated factors	8	High
20	Tsai et al. (2020) (46)	Taiwan, China	Single institution; curative OSCC surgery; Jan 2014–Dec 2018	Retrospective cohort	OSCC	44/70	62.9	PG-SGA B/C	Unadjusted prevalence; no multivariable-adjusted estimate reported.	PG-SGA prevalence only; not included in associated-factor analyses.	6	Moderate

**Table 1** presents abbreviated assessment definitions for readability. Detailed study-specific thresholds, operational case definitions, assessment time points, and source-reported classifications are provided in **Supplementary Table 1**.

HNC, head and neck cancer; NPC, nasopharyngeal carcinoma; LAHNSCC, laryngeal and hypopharyngeal squamous cell carcinoma; SCC, squamous cell carcinoma; RT, radiotherapy; GLIM, Global Leadership Initiative on Malnutrition; PG-SGA, Patient-Generated Subjective Global Assessment; NRS 2002, Nutritional Risk Screening 2002; NOS, Newcastle-Ottawa Scale; AHRQ, Agency for Healthcare Research and Quality. Quality assessment: high quality (≥7 for NOS; ≥8 for AHRQ), moderate quality (4–6 for NOS; 4–7 for AHRQ), low quality (<4 for either instrument).

Cohort independence was assessed by comparing author groups, institutions, recruitment dates, eligibility criteria, participant counts, and event counts. For multiple reports from the same or substantially overlapping cohort, the most complete report was designated as the primary source and only one estimate per cohort–outcome–factor combination was eligible for a conventional meta-analysis. For longitudinal studies, the earliest eligible assessment was prespecified for the primary prevalence analysis; later correlated time points were retained for descriptive or sensitivity analyses only. Multilevel or robust-variance methods were to be used only if more than one correlated estimate from a cohort was intentionally retained.

### 2.4 Risk-of-bias and methodological quality assessment

The risk of bias of included studies was assessed independently by two reviewers using domain-based tools matched to the purpose of each synthesis. For prevalence analyses, the Joanna Briggs Institute (JBI) critical appraisal checklist for prevalence studies was used because these analyses focused on the frequency of malnutrition, nutritional risk, or PG-SGA-based nutritional categories (20, 21). The assessed domains included sampling frame, sampling method, sample size, description of study participants and setting, data analysis, validity of outcome identification, reliability of measurement, statistical analysis, and response rate or completeness of data.

For associated-factor analyses, the Quality in Prognosis Studies (QUIPS) tool was applied because the review synthesized variables associated with malnutrition or nutritional risk (22). The QUIPS assessment covered six domains: study participation, study attrition, measurement of the associated factor, measurement of the nutritional outcome, adjustment for confounding, and statistical analysis and reporting. Each domain was judged as low, moderate, high, or unclear risk of bias. Particular attention was given to confounding and temporal bias, because several included studies were cross-sectional or reported baseline associations, and many estimates were unadjusted or adjusted for different covariate sets.

The Newcastle-Ottawa Scale (NOS) and AHRQ checklist were retained as descriptive methodological-quality references to preserve comparability with the original extraction framework (23, 24); however, total scores were not used as the primary basis for judging risk of bias. Overall judgments were based on the domain-level JBI and QUIPS assessments rather than on numerical scores alone. Disagreements between reviewers were resolved by discussion or adjudication by a third reviewer. Domain-level judgments and the rationale for each included study were reported in **Supplementary Table 3**. The certainty of evidence for the main pooled outcomes was summarized narratively by considering risk of bias, inconsistency, indirectness, imprecision, and small-study or publication-bias concerns, consistent with the GRADE framework (25).

### 2.5 Statistical methods

All statistical analyses were performed using Stata version 18.0 (StataCorp LLC, College Station, TX, USA) and independently verified in R using the meta and metafor packages. Separate proportion meta-analyses were conducted according to the nutritional-outcome construct: (1) prevalence of diagnostically defined malnutrition, primarily GLIM-defined malnutrition; (2) prevalence of nutritional risk, primarily NRS 2002-defined nutritional risk; and (3) prevalence based on PG-SGA nutritional categories or study-defined PG-SGA cut-offs. Random-effects models were specified *a priori* because heterogeneity was expected across cancer subsites, treatment phases, geographic settings, study designs, and outcome definitions; model selection was therefore not based on the Cochran Q test or I² threshold. Between-study variance (τ²) was estimated by restricted maximum likelihood (REML). For prevalence, the primary analysis used a logit transformation under a random-effects model with REML estimation (26). The Freeman–Tukey double-arcsine transformation was applied as a sensitivity analysis because its inverse back-transformation may be affected by differences in study size (27). Pooled prevalence was planned only for prospective cohorts in which participants were free of the specified outcome at baseline and new cases were observed during follow-up; no included report met this definition.

Associated-factor estimates were pooled only when the nutritional outcome construct, factor definition, reference category, and temporal classification were clinically comparable. Estimates with clearly antecedent factor measurement were classified as prospective predictors, whereas concurrent or temporally unclear estimates were classified as correlates or clinical markers. Variables incorporated into the outcome definition were not treated as independent predictors. In particular, low BMI was interpreted cautiously because BMI is a phenotypic component of GLIM-defined malnutrition; therefore, low-BMI estimates were not pooled as evidence of an independent antecedent predictor when GLIM-defined outcomes could not be separated from the exposure definition. Similarly, albumin, hemoglobin, dysphagia, and tube feeding were interpreted according to their temporal and clinical role: albumin and hemoglobin were considered concurrent biochemical or clinical markers unless measured before malnutrition assessment; dysphagia was considered a functional marker and potential intermediate mechanism; and prolonged tube feeding was considered susceptible to reverse causality and confounding by indication because enteral support is often initiated after nutritional vulnerability or impaired oral intake has already become clinically apparent.

Adjusted and unadjusted estimates were not pooled indiscriminately. When at least three studies reported adjusted ORs for the same associated variable with comparable definitions, reference categories, and outcome constructs, adjusted estimates were used for the primary pooled analysis. Unadjusted estimates were analyzed separately or summarized descriptively when the number of studies was insufficient for a separate meta-analysis. The adjustment status, adjusted covariates, reference category, variable cut-off, exposure timing, outcome timing, and effect-measure type were recorded at the study level. ORs, RRs, and HRs were not combined in the same pooled estimate unless a justified conversion was possible. Continuous variables dichotomized using substantially different thresholds were not pooled as identical exposures and were instead interpreted descriptively or examined in sensitivity analyses where appropriate. For study-level proportion estimates, the observed prevalence was calculated as the number of events divided by the corresponding analysis denominator and was transformed to the logit scale for the primary random-effects analysis. For association estimates reported as odds ratios, the natural logarithm of the OR was analyzed, and its standard error was derived from the reported 95% confidence interval as [ ln(upperCI)−ln(lowerCI)]/3.92 when a standard error was not directly available. The calculation procedures, transformations, heterogeneity statistics, and multiplicity adjustment used in the quantitative synthesis are summarized in **Supplementary Table 6**.

Sensitivity analyses included sequential study exclusion and a modified Hartung–Knapp small-sample correction for each associated-factor synthesis. The modified correction constrained the variance inflation factor to be no smaller than 1, thereby preventing confidence intervals from becoming narrower than the conventional random-effects interval. Analyses based on three studies were treated as exploratory, and 95% prediction intervals were reported only when at least four studies were available and the interval was estimable. Additional stratification by adjustment status, outcome definition, and temporal classification was undertaken only when sufficient comparable studies were available. Publication-bias assessment was prespecified for analyses with at least 10 contributing studies; because no synthesis met that threshold, funnel plots, Egger tests, and trim-and-fill procedures were not performed.

All statistical tests were two-tailed. For every associated-factor synthesis, the P value for the pooled effect ( $P_{effect}$), the Cochran Q-test P value for heterogeneity ( $P_{heterogeneity}$), and the Benjamini–Hochberg false-discovery-rate-adjusted q value were calculated and reported separately. The Benjamini–Hochberg procedure was applied across the 10 associated-factor comparisons, with q<0.05 used as the multiplicity-adjusted threshold (28). Modified Hartung–Knapp P values were reported separately as sensitivity results and were not substituted for the primary REML effect-test P values.

## 3 Results

### 3.1 Literature search results

The updated searches identified 6,343 records from bibliographic databases and 96 additional records from citation searching and guideline sources, yielding 6,439 records before deduplication. After deduplication in EndNote X9 and manual verification, 1,712 duplicate records were removed, leaving 4,727 unique records for title and abstract screening. Of these, 4,418 records were excluded because they did not meet the population, outcome, exposure, or study-design criteria. A total of 309 reports were sought for retrieval; 12 reports could not be retrieved despite database, library, and web searches. Therefore, 297 full-text reports were assessed for eligibility.

**Figure 1:**
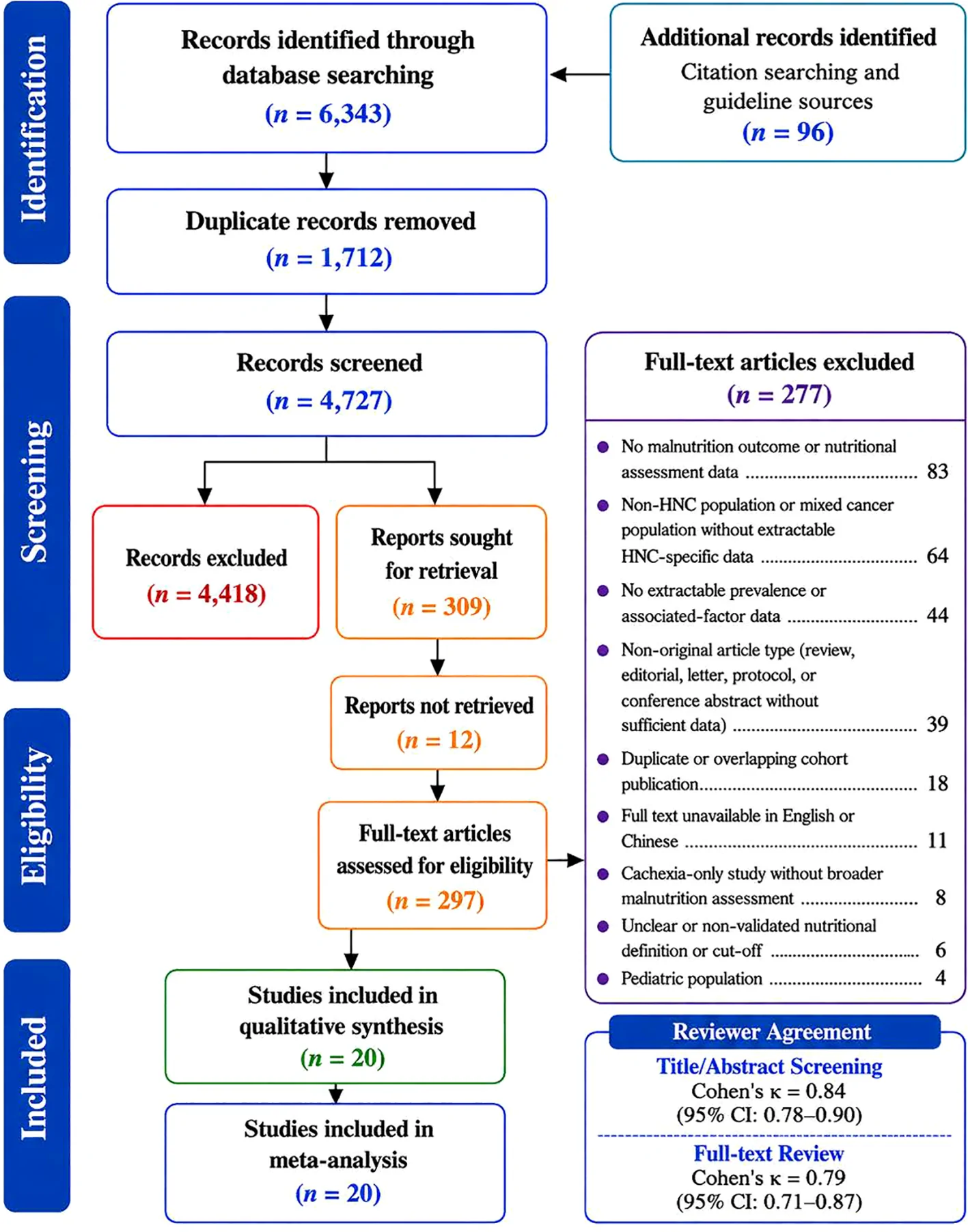
PRISMA 2020 flow diagram of study selection for the systematic review and meta-analysis of factors associated with malnutrition in head and neck cancer.

A total of 277 full-text reports were excluded for mutually exclusive reasons: no malnutrition outcome or nutritional assessment data (n=83), non-HNC population or mixed cancer population without extractable HNC-specific data (n=64), no extractable prevalence or associated-factor data (n=44), non-original article type such as review, editorial, letter, protocol, or conference abstract without sufficient data (n=39), duplicate or overlapping cohort publication (n=18), full text unavailable in English or Chinese (n=11), pediatric population (n=4), cachexia-only study without broader malnutrition assessment (n=8), and unclear or non-validated nutritional definition or cut-off (n=6). Finally, 20 studies were included in the qualitative synthesis and quantitative meta-analysis. The reconstructed PRISMA 2020 flow diagram is presented in **Figure 1**.

The inter-rater agreement for title and abstract screening was excellent (Cohen’s kappa=0.84; 95% CI: 0.78–0.90), and inter-rater consistency was also good for full-text review (Cohen’s kappa=0.79; 95% CI: 0.71–0.87).

### 3.2 Characteristics and quality of included studies

After completion of the reference, cohort-independence, and data-traceability audit, the final evidence set comprised 20 studies with a summed reported sample size of 4,456 patients with HNC, published between 2020 and 2026. Twelve studies were conducted in mainland China and one in Taiwan, China. Three studies were conducted in Europe, including Sweden, Finland, and Spain, and one study each was conducted in Australia, Morocco, Brazil, and India. When the study designs were grouped into broad methodological categories, eight studies were cross-sectional (40.0%), four were prospective cohort studies (20.0%), three were retrospective cohort studies (15.0%), and three were longitudinal studies (15.0%). One study was a secondary analysis of a randomized controlled trial (5.0%), and one was an observational study for which a more specific design classification was not reported (5.0%).

The study populations included patients with HNC or HNSCC across multiple head and neck sites in 10 studies (50.0%), patients with nasopharyngeal carcinoma in five studies (25.0%), patients with oral cancer or oral squamous cell carcinoma in four studies (20.0%), and patients with laryngeal or hypopharyngeal squamous cell carcinoma in one study (5.0%). The principal nutritional outcome measures were NRS 2002, GLIM, and PG-SGA, each used in six studies. Of the remaining two studies, one evaluated a source-defined prognostic nutritional index trajectory, and one used a model-defined malnutrition outcome rather than one of the three principal assessment instruments.

To improve transparency, **Table 1** summarizes the core characteristics of the included studies, including the study design, population, sample size, nutritional assessment instrument, case definition or cut-off, assessment time point, and event count. **Supplementary Table 1** provides the corresponding study-level evidence traceability and extraction details, including the full citation and DOI/PMID or database identifier, recruitment setting and period, extracted effect estimate and reference category, adjustment status and covariates, exact meta-analysis contribution, and cohort-overlap or independence decision for each study record. The original NOS and AHRQ scores were retained in **Table 1** only as descriptive indicators of general methodological reporting quality and were not used as the sole basis for judging risk of bias. A more detailed domain-level appraisal was therefore performed and is presented in **Supplementary Table 3**. For prevalence estimates, the JBI checklist identified generally acceptable outcome-measurement domains because most studies used established tools such as GLIM, PG-SGA, or NRS 2002; however, several studies showed moderate concerns regarding sampling representativeness, response-rate reporting, or completeness of data. For associated-factor analyses, the QUIPS assessment showed that confounding control and temporal sequence were the most frequent concerns, particularly in cross-sectional or baseline analyses. Therefore, although no study was excluded solely on the basis of methodological appraisal, the quality categories should not be interpreted as evidence of uniformly low risk of bias.

### 3.3 Meta-analysis results

#### 3.3.1 Prevalence by nutritional outcome construct

As shown in **Table 2**, the pooled prevalence was highest for the PG-SGA-based nutritional category at 57.8%, followed by GLIM-defined malnutrition at 39.4% and NRS 2002-defined nutritional risk at 32.0%. The point estimates from logit scale REML and Freeman-Tukey analyses were highly consistent, with overlapping confidence intervals, indicating that the primary study outcome was insensitive to the conversion method. However, the high I² values and wide prediction intervals suggest considerable differences among studies in patient characteristics, treatment stage, and clinical setting. Therefore, these estimates should be interpreted separately rather than as interchangeable measures of malnutrition. No pooled incidence estimate was calculated because none of the included studies documented new cases among participants confirmed to be free of the specified nutritional outcome at baseline.

**Table 2:** Pooled prevalence by nutritional outcome construct and assessment instrument.

Outcome construct/assessment instrument	Studies	Total N	Logit REML pooled rate, % (95% CI)	τ²	I²	P (heterogeneity)	95% prediction interval, %	Freeman–Tukey sensitivity, % (95% CI)
GLIM-defined malnutrition	5	1,158	39.4 (30.4–49.1)	0.170	89.7%	<0.001	13.1-73.7	39.6 (30.5–49.0)
PG-SGA-based nutritional category	6	724	57.8 (37.7–75.7)	0.982	96.7%	<0.001	6.5-96.4	57.8 (39.0–75.4)
NRS 2002-defined nutritional risk	6	1,423	32.0 (18.9–48.7)	0.741	94.9%	<0.001	3.4-86.3	33.3 (20.2–47.8)

GLIM, Global Leadership Initiative on Malnutrition; PG-SGA, Patient-Generated Subjective Global Assessment; NRS 2002, Nutritional Risk Screening 2002; CI, confidence interval. GLIM was treated as a diagnostic malnutrition framework, PG-SGA as an oncology-oriented nutritional assessment/classification tool, and NRS 2002 as a nutritional-risk screening tool.

**Figure 2:**
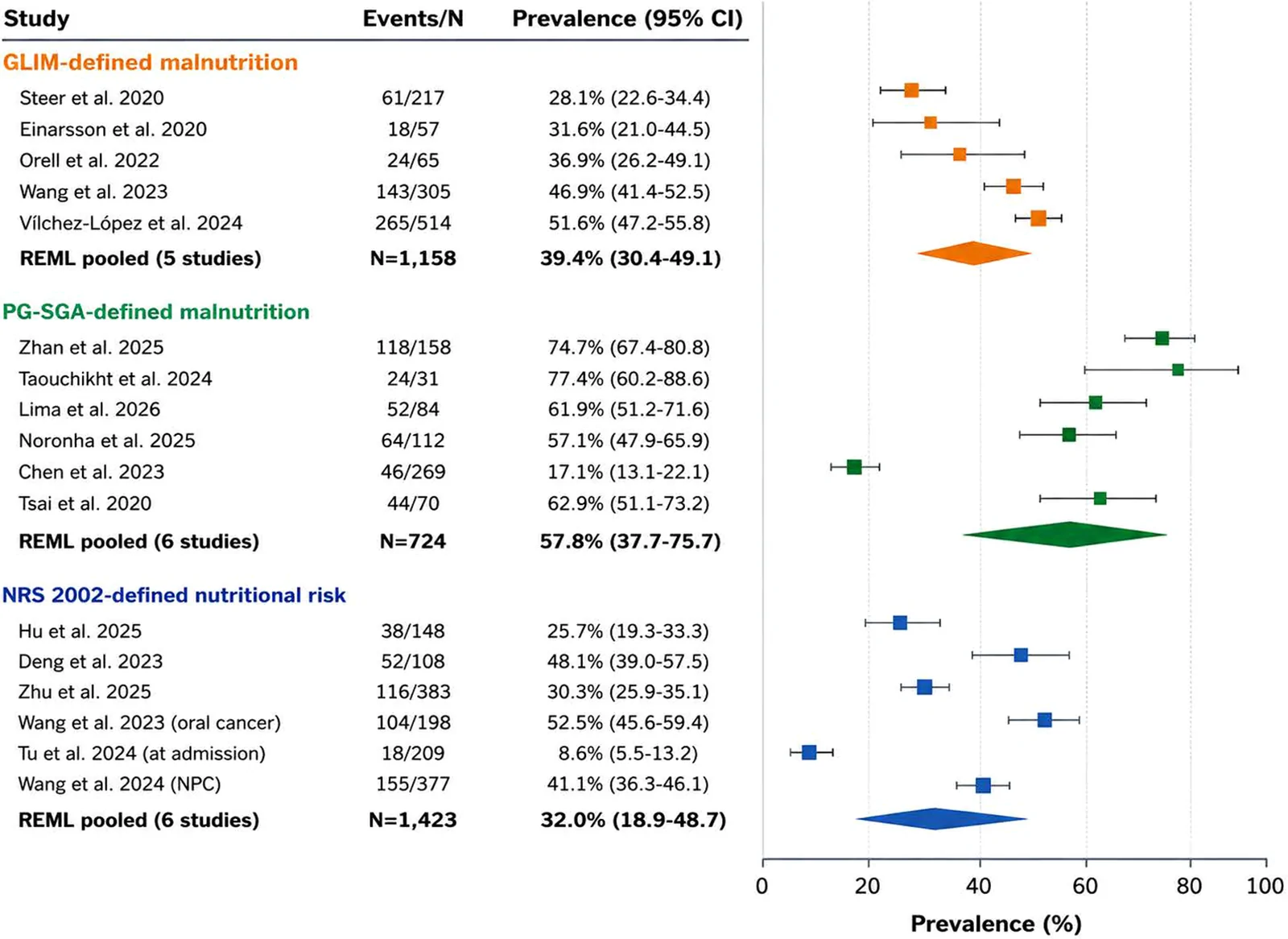
REML forest plot based on logit scale, showing the prevalence estimates stratified by nutritional outcome measures. Squares represent specific proportions from each study, and diamonds represent pooled estimates. CI, confidence interval.

These findings confirm that PG-SGA, NRS 2002, and GLIM should not be interpreted as interchangeable diagnostic tools. PG-SGA captures oncology-oriented symptom burden, intake limitation, and functional deterioration; NRS 2002 identifies nutritional risk; and GLIM provides a diagnostic framework for malnutrition. Therefore, the pooled estimates are reported by outcome construct rather than as a single interchangeable malnutrition prevalence estimate. The logit and Freeman–Tukey analyses yielded closely aligned point estimates, but the broad prediction intervals showed that prevalence can vary markedly across clinical settings. The forest plot depicting study-specific and pooled prevalence estimates stratified by assessment instrument and outcome construct is presented in **Figure 2**.

#### 3.3.2 Factors associated with malnutrition

##### 3.3.2.1 General factors

**Figure 3:**
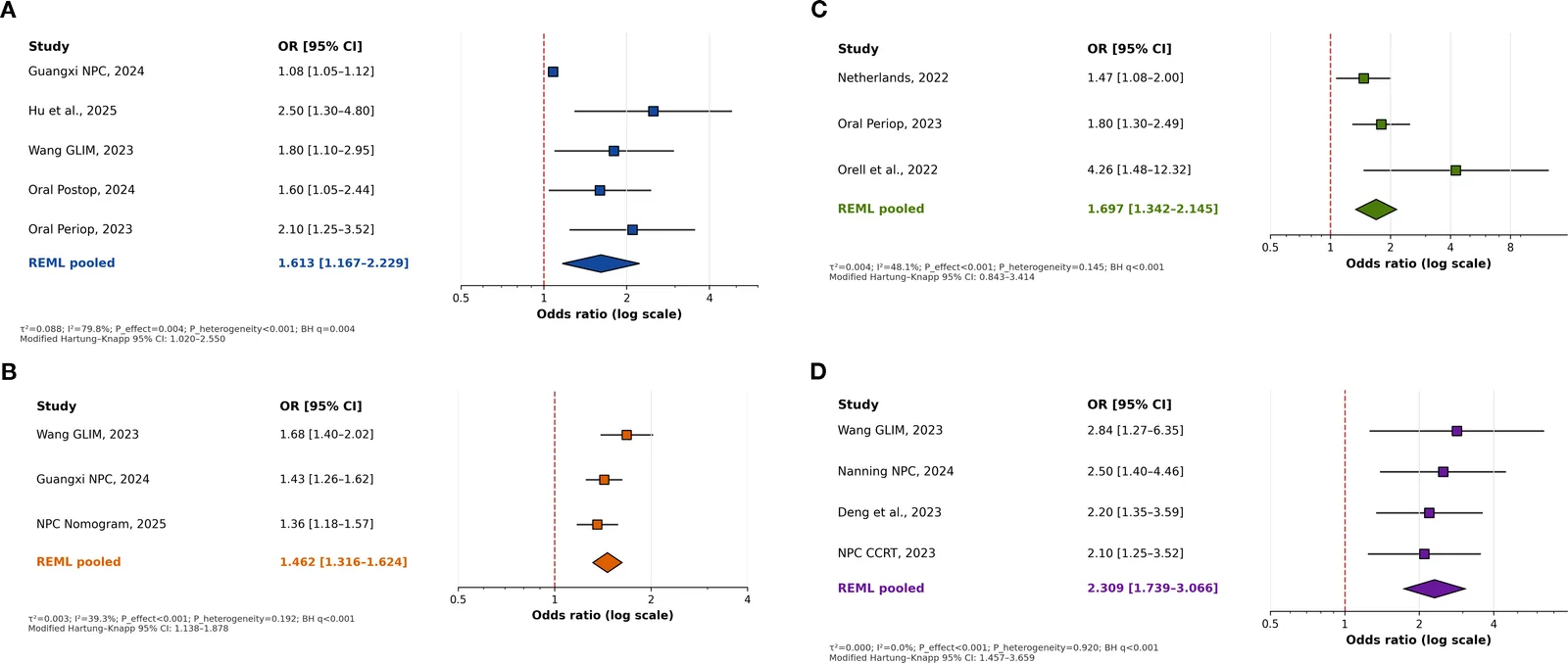
**(A)** Recalculated REML forest plot. Forest plot of the association between advanced age and malnutrition risk in head and neck cancer. OR, odds ratio; CI, confidence interval. **(B)** REML forest plot of the association between low body mass index and malnutrition in head and neck cancer. **(C)** Recalculated REML forest plot. Forest plot of the association between smoking and malnutrition in head and neck cancer. **(D)** Recalculated REML forest plot. Forest plot of the association between anxiety or depression and malnutrition in head and neck cancer.

Advanced age was examined in five studies (n=1,231). The REML estimate indicated higher odds of malnutrition (OR = 1.613, 95% CI: 1.167–2.229; $P_{effect}$ =0.004; q=0.004), with substantial heterogeneity (τ²=0.088; I²=79.8%; $P_{heterogeneity}$ <0.001). The modified Hartung–Knapp sensitivity interval was wider (95% CI: 1.020–2.550; P = 0.044), and the 95% prediction interval included the null (0.547–4.754). The forest plot for this analysis is presented in **Figure 3A**.

Low body mass index (BMI) was assessed in three studies (n=997). The recalculated REML estimate was OR = 1.462 (95% CI: 1.316–1.624; $P_{effect}$ <0.001; q<0.001), with τ²=0.003, I²=39.3%, and $P_{heterogeneity}$ =0.192. The modified Hartung–Knapp sensitivity interval remained above the null (95% CI: 1.138–1.878; P = 0.023). Because low BMI is also a phenotypic criterion within GLIM-based malnutrition diagnosis, this association may partly reflect incorporation bias when GLIM-defined outcomes were used. Therefore, low BMI was interpreted as a concurrent anthropometric marker of nutritional compromise rather than as an antecedent causal factor. The forest plot is presented in **Figure 3B**.

Smoking was evaluated in three studies (n=493). The primary REML estimate was OR = 1.697 (95% CI: 1.342–2.145; $P_{effect}$ <0.001; q<0.001), with τ²=0.004, I²=48.1%, and $P_{heterogeneity}$ =0.145. Under the modified Hartung–Knapp sensitivity analysis, the confidence interval crossed the null (95% CI: 0.843–3.414; P = 0.083); this association was therefore considered less robust. The forest plot is presented in **Figure 3C**.

Anxiety and/or depression was examined in four studies (n=781). The REML estimate was OR = 2.309 (95% CI: 1.739–3.066; $P_{effect}$ <0.001; q<0.001), with τ²=0, I²=0%, and $P_{heterogeneity}$ =0.920. The association remained statistically significant with the modified Hartung–Knapp correction (95% CI: 1.457–3.659; P = 0.010), and the 95% prediction interval was 1.239–4.303. The forest plot is presented in **Figure 3D**.

##### 3.3.2.2 Biochemical and clinical factors

**Figure 4:**
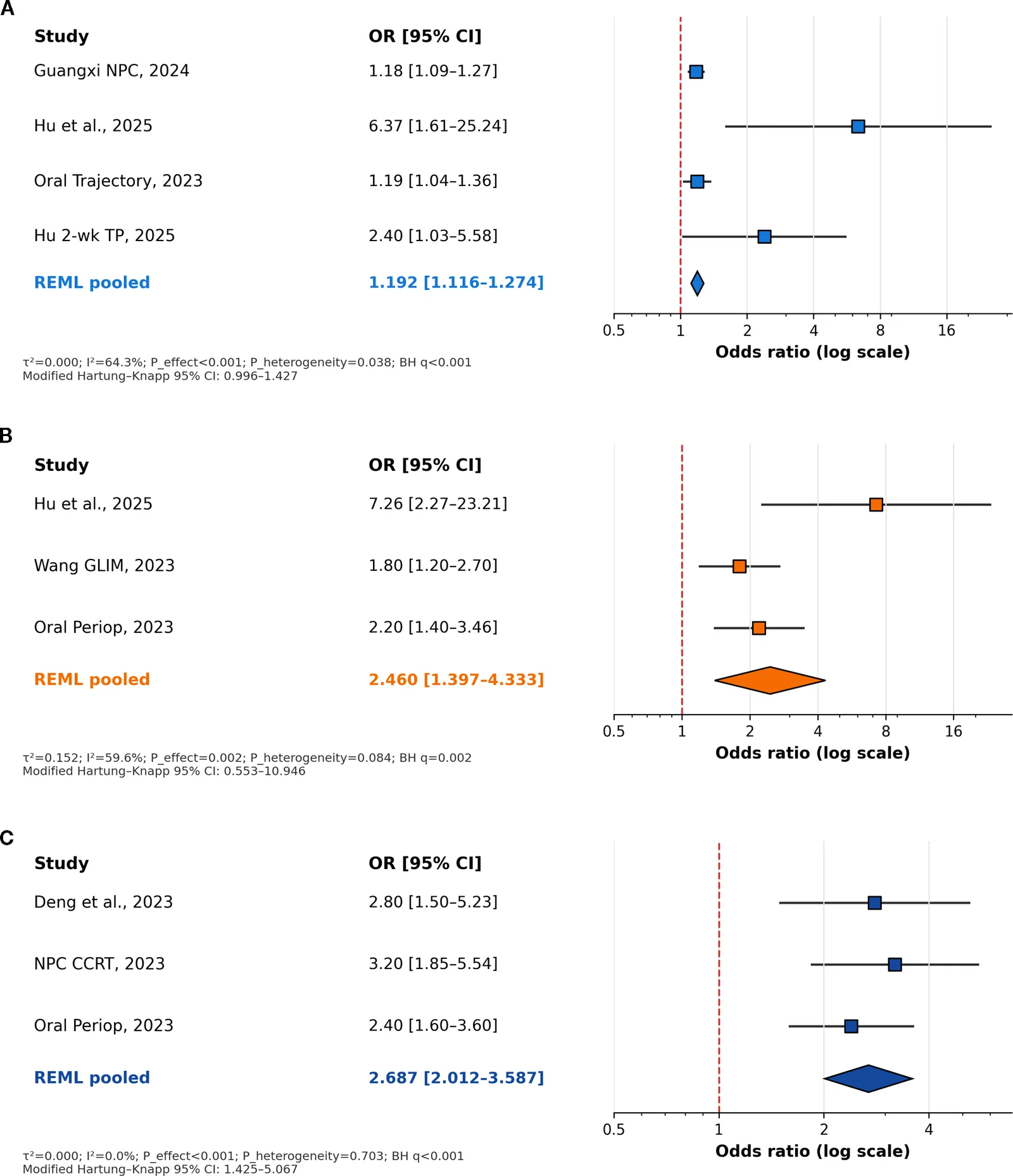
**(A)** REML forest plot of the association between low serum albumin and malnutrition in head and neck cancer. **(B)** REML forest plot of the association between low hemoglobin and malnutrition in head and neck cancer. **(C)** REML forest plot of the association between dysphagia and malnutrition in head and neck cancer.

Low serum albumin was reported in four studies (n=1,372). The primary REML estimate was OR = 1.192 (95% CI: 1.116–1.274; $P_{effect}$ <0.001; q<0.001), with τ²=0, I²=64.3%, and $P_{heterogeneity}$ =0.038. The modified Hartung–Knapp interval included the null (95% CI: 0.996–1.427; P = 0.053), and the 95% prediction interval was 0.935–1.520. Because albumin is a negative acute-phase protein, this result should be interpreted as an association with clinical and inflammatory status rather than as evidence of an independent causal effect. The forest plot is presented in **Figure 4A**.

Low hemoglobin was evaluated in three studies (n=524 patients). Low hemoglobin was associated with higher odds of malnutrition. The primary REML estimate was OR = 2.460 (95% CI: 1.397–4.333; $P_{effect}$ =0.002; q=0.002), with τ²=0.152, I²=59.6%, and $P_{heterogeneity}$ =0.084. The modified Hartung–Knapp interval was wide and crossed the null (95% CI: 0.553–10.946; P = 0.122), indicating substantial small-sample imprecision. This finding was therefore interpreted cautiously as a clinical marker that may reflect inflammation, bleeding, deficiency, marrow suppression, treatment toxicity, or malnutrition itself. The forest plot is presented in **Figure 4B**.

Dysphagia was examined in three studies (n=499 patients). Swallowing difficulty was associated with higher odds of malnutrition. The REML estimate was OR = 2.687 (95% CI: 2.012–3.587; $P_{effect}$ <0.001; q<0.001), with τ²=0, I²=0%, and $P_{heterogeneity}$ =0.703. The association remained statistically significant under the modified Hartung–Knapp sensitivity analysis (95% CI: 1.425–5.067; P = 0.022). Given its close relationship with tumor site, disease extent, and treatment toxicity, dysphagia was interpreted as an intermediate functional mechanism and clinical warning marker rather than as an independent etiological factor. The forest plot is presented in **Figure 4C**.

##### 3.3.2.3 Disease-related and treatment-related factors

**Figure 5:**
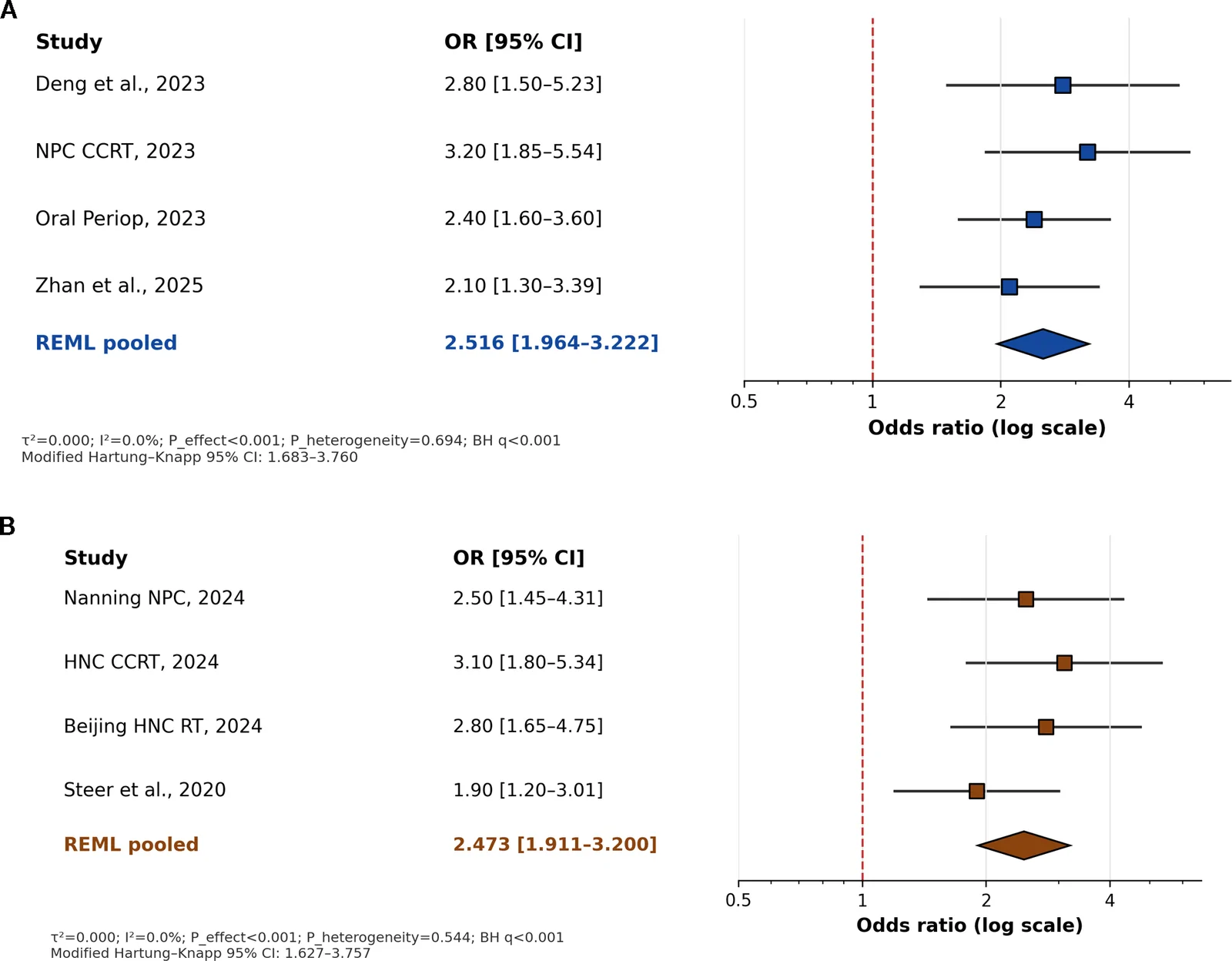
**(A)** REML forest plot of the association between advanced tumor stage (III–IV) and malnutrition in head and neck cancer. **(B)** REML forest plot of the association between radiotherapy or chemoradiotherapy and malnutrition in head and neck cancer.

Advanced tumor stage (III–IV) was evaluated in four studies (n=1,020). The REML estimate was OR = 2.516 (95% CI: 1.964–3.222; $P_{effect}$ <0.001; q<0.001), with τ²=0, I²=0%, and $P_{heterogeneity}$ =0.694. The modified Hartung–Knapp interval remained above the null (95% CI: 1.683–3.760; P = 0.005), and the 95% prediction interval was 1.461–4.332. This finding was interpreted as a marker of greater disease burden and clinical complexity because staging is closely linked to tumor burden, dysphagia, pain, treatment intensity, and systemic inflammation. The forest plot is presented in **Figure 5A**.

Radiotherapy or chemoradiotherapy was examined in four studies (n=901). Patients undergoing radiotherapy or concurrent chemoradiotherapy had higher odds of malnutrition. The REML estimate was OR = 2.473 (95% CI: 1.911–3.200; $P_{effect}$ <0.001; q<0.001), with τ²=0, I²=0%, and $P_{heterogeneity}$ =0.544. The association remained statistically significant with the modified Hartung–Knapp correction (95% CI: 1.627–3.757; P = 0.006), and the 95% prediction interval was 1.404–4.354. The magnitude may nevertheless depend on treatment dose, irradiated volume, regimen, nutritional support, assessment timing, and baseline disease severity. The forest plot is presented in **Figure 5B**.

Prolonged tube feeding was assessed in three studies (n=341). The primary REML estimate was not statistically significant (OR = 1.485, 95% CI: 0.911–2.419; $P_{effect}$ =0.112; q=0.112), with τ²=0.152, I²=81.4%, and $P_{heterogeneity}$ =0.005. The modified Hartung–Knapp sensitivity interval was wider (95% CI: 0.508–4.338; P = 0.253). This result should not be interpreted as evidence that tube feeding increases malnutrition risk because reverse causality and confounding by indication are likely. The wide confidence interval and high heterogeneity also suggest variation in the timing, indication, and duration of enteral support across studies. The pooled results of all associated factors are summarized in **Table 3**.

**Table 3:** Pooled results of factors associated with malnutrition in head and neck cancer.

Category	Associated factor	Studies	REML OR (95% CI)	$\tau^{2}$	${\text{I}}^{2}$	$P_{heterogeneity}$	$P_{effect}$	BH q
General	Advanced age	5	1.613 (1.167–2.229)	0.088	79.8%	<0.001	0.004	0.004
General	Low BMI	3	1.462 (1.316–1.624)	0.003	39.3%	0.192	<0.001	<0.001
General	Smoking	3	1.697 (1.342–2.145)	0.004	48.1%	0.145	<0.001	<0.001
General	Anxiety/Depression	4	2.309 (1.739–3.066)	0.000	0.0%	0.920	<0.001	<0.001
Biochemical	Low albumin	4	1.192 (1.116–1.274)	0.000	64.3%	0.038	<0.001	<0.001
Biochemical	Low hemoglobin	3	2.460 (1.397–4.333)	0.152	59.6%	0.084	0.002	0.002
Biochemical	Dysphagia	3	2.687 (2.012–3.587)	0.000	0.0%	0.703	<0.001	<0.001
Disease-related	Advanced stage (III–IV)	4	2.516 (1.964–3.222)	0.000	0.0%	0.694	<0.001	<0.001
Disease-related	Radiotherapy/Chemoradiotherapy	4	2.473 (1.911–3.200)	0.000	0.0%	0.544	<0.001	<0.001
Disease-related	Prolonged tube feeding	3	1.485 (0.911–2.419)	0.152	81.4%	0.005	0.112	0.112

OR, odds ratio; CI, confidence interval; BMI, body mass index; REML, restricted maximum likelihood; BH, Benjamini–Hochberg. $P_{effect}$ tests the pooled association; $P_{heterogeneity}$ is the Cochran Q-test P value; q is the BH false-discovery-rate-adjusted P value across 10 associated-factor comparisons.

### 3.4 Sensitivity analysis

**Figure 6:**
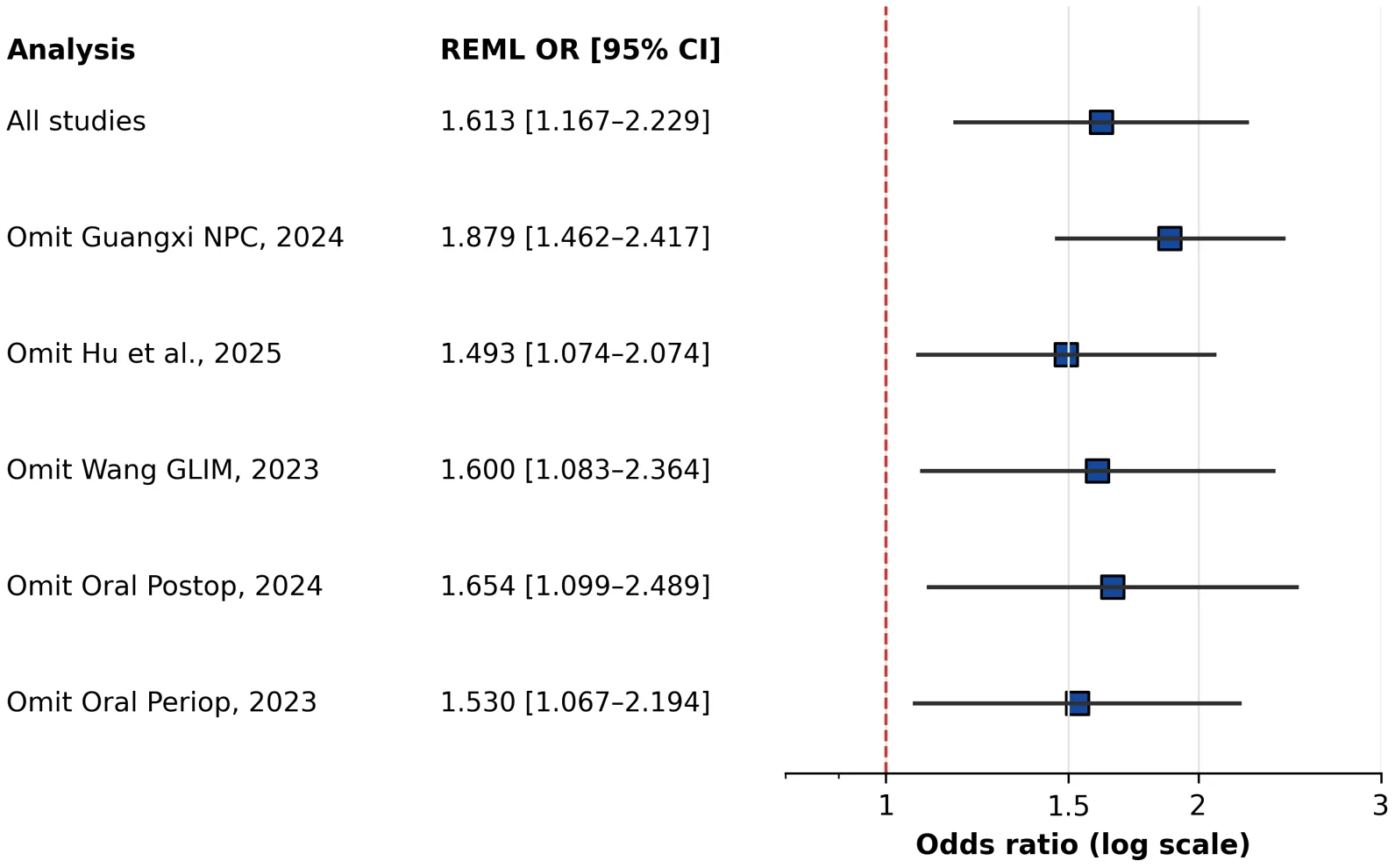
Recalculated leave-one-out REML sensitivity analysis for advanced age. All conventional REML confidence intervals remain above the null, although the modified Hartung–Knapp interval and prediction interval are wider.

To assess the robustness of the pooled estimates, sensitivity analyses were performed by sequentially excluding individual studies from the meta-analysis of advanced age. After individual exclusion analysis for advanced age, the REML OR values ranged as follows: 1.493 (95% CI: 1.074–2.074) after excluding the study by Hu et al., and 1.879 (95% CI: 1.462–2.417) after excluding the Guangxi nasopharyngeal carcinoma study. All recalculated conventional REML confidence intervals were above 1.0. These findings confirm that the pooled estimate for advanced age was not driven by any single influential study. The sensitivity analysis results for the advanced-age association are presented in **Figure 6**.

However, the wide prediction intervals and the revised Hartung-Knapp results warrant careful interpretation. This study also performed a revised Hartung-Knapp sensitivity analysis on all 10 associated factors. Advanced age, low BMI, anxiety/depression, dysphagia, advanced stage, and radiotherapy or chemoradiotherapy remained nominally statistically significant. Smoking, low albumin, and low hemoglobin crossed null values after small sample correction, while long-term tube feeding remained statistically insignificant. The analysis based on the three studies was considered exploratory, therefore the prediction intervals for these estimates were omitted. The factor-specific modified Hartung–Knapp estimates and prediction intervals are presented in **Table 4**, while the broader sensitivity-analysis framework and robustness findings across prevalence and associated-factor syntheses are summarized in **Supplementary Table 7**.

**Table 4:** Modified Hartung–Knapp sensitivity analyses and prediction intervals.

Associated factor	REML OR	Modified Hartung–Knapp 95% CI	$P_{MHK}$	95% prediction interval
Advanced age	1.613	1.020–2.550	0.044	0.547–4.754
Low BMI	1.462	1.138–1.878	0.023	Not reported (k=3)
Smoking	1.697	0.843–3.414	0.083	Not reported (k=3)
Anxiety/depression	2.309	1.457–3.659	0.010	1.239–4.303
Low albumin	1.192	0.996–1.427	0.053	0.935–1.520
Low hemoglobin	2.460	0.553–10.946	0.122	Not reported (k=3)
Dysphagia	2.687	1.425–5.067	0.022	Not reported (k=3)
Advanced stage (III–IV)	2.516	1.683–3.760	0.005	1.461–4.332
Radiotherapy/chemoradiotherapy	2.473	1.627–3.757	0.006	1.404–4.354
Prolonged tube feeding	1.485	0.508–4.338	0.253	Not reported (k=3)

Modified Hartung–Knapp intervals use a variance inflation factor constrained to be at least 1. Prediction intervals were reported only for analyses with at least four studies; three-study analyses were treated as exploratory.

### 3.5 Publication bias assessment

Formal publication-bias analyses were not performed. According to conventional meta-analytic practice, funnel plots and Egger’s linear regression test are generally recommended only when a pooled analysis includes at least 10 studies, because tests for asymmetry are underpowered and potentially misleading with smaller numbers of studies. In the present review, the assessment tool-specific prevalence analyses included 5, 6, and 6 studies, respectively, and all individual associated-factor analyses included between 3 and 5 studies. Therefore, no outcome met the prespecified threshold for publication-bias testing. This decision was consistent with the statistical analysis plan and avoids overinterpretation of small-sample funnel-plot asymmetry.

## 4 Discussion

### 4.1 Principal findings and clinical meaning

This systematic review and meta-analysis have combined the data of 20 studies on 4,456 patients with head and neck cancer (HNC). Prevalence estimates were different in the GLIM, PG-SGA and NRS 2002 constructs, and wide prediction intervals showed significant variation among clinical environments. The first REML analyses found some related clinical factors; however, the modified Hartung-Knapp sensitivity analysis showed that the estimates for smoking, low albumin and low hemoglobin remained imprecise due to a lack of studies. The risk-of-bias assessment did not find any significant methodological deficiencies that required excluding the study. However, the domain-level evaluation also showed some moderate problems in several studies, such as the representativeness of the sample, control of confounding factors, time sequence, and statistical adjustments. Therefore, the pooled results should be regarded as clinically meaningful summaries of prevalence and association, not as high-certainty evidence of causality.

Compared with the 2024 review by Wang et al. (11), which searched four English-language databases through June 2023 and narratively summarized 18 studies involving 2,593 participants, the present review searched nine databases through 4 July 2026 and included 20 studies involving 4,456 participants. Study-level cross-checking identified three overlapping reports and 17 studies not included in the earlier review. The expanded search included CNKI, Wanfang, VIP, and CBM/SinoMed, and 11 of the 20 included studies were conducted in China, improving representation of Chinese clinical settings. The present review also separated GLIM-defined malnutrition, PG-SGA categories, and NRS 2002-defined nutritional risk and quantitatively pooled tool-specific prevalence and clinically comparable associated-factor estimates with heterogeneity, prediction-interval, and sensitivity analyses. It should therefore be regarded as an updated, multilingual quantitative extension of the 2024 narrative synthesis rather than the first systematic review of malnutrition-associated factors in HNC.

The pooled estimates indicate that malnutrition and nutritional risk are common across the HNC trajectory, although their frequency varies substantially by assessment instrument, treatment phase, and clinical setting. The largest point estimates involved dysphagia, advanced tumor stage, radiotherapy or chemoradiotherapy, anxiety or depression, and low hemoglobin; however, these analyses generally included few studies, and several sensitivity intervals were imprecise. These variables may reflect impaired intake, disease and treatment burden, or reduced psychological and physiological reserve. Given the observational and partly cross-sectional evidence base, they should be interpreted as associated clinical markers rather than independent causal determinants.

### 4.2 Interpretation of differences across nutritional assessment tools

The variation in pooled prevalence across GLIM, PG-SGA, and NRS 2002 should not be interpreted simply as disagreement among tools. These instruments answer different clinical questions. NRS 2002 is primarily a screening instrument designed to identify patients who may benefit from nutritional intervention; PG-SGA captures symptom burden, intake limitation, and functional change in an oncology-specific manner; and GLIM provides a diagnostic framework that requires both phenotypic and etiologic evidence of malnutrition (13, 14, 47). Therefore, PG-SGA may identify a broader group of patients with active nutritional deterioration, whereas GLIM may classify a more diagnostically specific subgroup and NRS 2002 should be interpreted as nutritional risk rather than a definitive malnutrition diagnosis (48). The practical implication is that HNC services should consider a two-step pathway: rapid screening at diagnosis and before treatment, followed by comprehensive assessment for patients with positive screening results or multiple associated clinical variables.

High heterogeneity in prevalence estimates also reflects real clinical differences. Patients assessed during radiotherapy, after surgery, or in advanced disease are exposed to different combinations of mucositis, xerostomia, odynophagia, dysgeusia, nausea, and swallowing dysfunction. Pooling these settings without considering treatment phase inevitably produces broad estimates. Future studies should report diagnostically defined malnutrition, nutritional risk, and PG-SGA categories separately at pre-treatment baseline, during active treatment, immediately after treatment, and during survivorship so that nutritional trajectories rather than single cross-sectional snapshots can be evaluated.

### 4.3 Mechanistic interpretation of associated factors

Dysphagia showed one of the largest pooled associations with malnutrition, but it should not be interpreted as an independent etiological factor. In HNC, dysphagia commonly results from tumor location and extent, surgical alteration of anatomy, radiation-induced fibrosis, mucosal injury, neuromuscular dysfunction, or the toxicity of radiotherapy and chemoradiotherapy. It is therefore better conceptualized as an intermediate functional mechanism linking disease burden and treatment effects to reduced oral intake. Its close correlation with tumor stage, therapeutic modality, and disease severity also raises the possibility of residual confounding. Clinically, dysphagia should be treated as an early warning marker for accelerated nutritional decline and should prompt timely swallowing and nutritional assessment.

Advanced tumor stage and radiotherapy or chemoradiotherapy were also associated with higher odds of malnutrition, but these findings require cautious interpretation. Advanced-stage disease may act as a marker of greater clinical complexity rather than as an independent causal exposure, because it is linked to larger tumor burden, anatomical involvement of the upper aerodigestive tract, pain, trismus, odynophagia, dysphagia, lower food intake, systemic inflammatory response, and more intensive treatment. Similarly, the association with radiotherapy or chemoradiotherapy may be influenced by treatment modality, total radiation dose, irradiated volume, chemotherapy regimen, timing of nutritional assessment, and use of nutritional support. Treatment-related adverse events, including oral mucositis, xerostomia, dysgeusia, odynophagia, dysphagia, nausea, and fatigue, represent intermediate mechanisms that progressively compromise intake (49, 50). Because the included studies did not uniformly control for these factors or examine mediation pathways, tumor stage and treatment modality should be interpreted as markers of disease burden and treatment toxicity rather than proven independent causes of malnutrition.

Psychological distress deserves particular attention because anxiety and depression were associated with more than twice the odds of malnutrition and showed no observed heterogeneity across contributing studies. Psychological distress can reduce appetite, motivation to prepare food, adherence to oral nutritional supplements, and engagement with rehabilitation. Conversely, malnutrition may worsen fatigue, sleep disturbance, cognitive function, and emotional resilience, reinforcing a bidirectional relationship (51). This supports the view that nutritional care in HNC should not be separated from psychosocial care.

The biochemical variables identified in this review should be interpreted as markers of clinical and inflammatory status rather than independent causal factors for malnutrition. Serum albumin is a negative acute-phase protein and may be influenced by systemic inflammation, the immune response to cancer, liver function, hydration status, clinical severity, and malnutrition itself. Low hemoglobin is similarly nonspecific because anemia in HNC may result from chronic inflammation, tumor bleeding, bone marrow suppression, micronutrient deficiency, antineoplastic treatment toxicity, or nutritional depletion. The strong clinical correlation between hypoalbuminemia, anemia, advanced tumor stage, and treatment intensity makes residual confounding likely. In practice, abnormal albumin or hemoglobin should prompt comprehensive nutritional evaluation rather than be used alone to define malnutrition or infer causality.

### 4.4 Implications for multidisciplinary clinical practice

The findings may inform a risk-stratified nutritional pathway for patients with HNC. Screening and comprehensive assessment may be considered at diagnosis, before major treatment, during active therapy, and during early follow-up, with frequency guided by relevant clinical guidance, institutional protocols, treatment phase, and individual risk rather than a fixed schedule derived from this meta-analysis. Patients presenting with dysphagia, advanced-stage disease, planned chemoradiotherapy, psychological distress, anemia, low BMI, older age (52), or smoking history (53) may warrant earlier dietitian-led assessment rather than waiting for clinically apparent weight loss.

Multidisciplinary care may be particularly appropriate for patients with multiple nutritional concerns. Dietitians can address energy and protein intake and oral nutritional supplementation; speech-language pathologists can assess swallowing and provide rehabilitation; oncology nurses can monitor intake, symptoms, weight trajectory, treatment toxicity, and adherence; physicians can manage pain, mucositis, nausea, xerostomia, and anemia; and psycho-oncology services can address anxiety, depression, and eating avoidance (54, 55). Coordinated care may help address nutritional decline, although the present observational synthesis does not establish the effect of any specific care model on treatment outcomes or survival.

The non-significant association between prolonged tube feeding and malnutrition requires careful interpretation. Tube feeding is usually initiated because a patient is already nutritionally vulnerable or unable to maintain oral intake; therefore, confounding by indication is likely. The observed heterogeneity suggests that studies varied in the timing, indication, and duration of tube feeding. Future work should distinguish prophylactic from reactive feeding strategies, nasogastric from gastrostomy approaches, and short-term treatment support from prolonged dependence. The key clinical question is not whether tube feeding is associated with malnutrition, but which patients benefit from earlier enteral support and how feeding can be combined with swallowing preservation (56).

### 4.5 Strengths and limitations

This review has several strengths. The search covered major English and Chinese databases and was supplemented by evidence resources and guideline repositories, reducing the risk of missing relevant studies. The inclusion of 4,456 patients improves precision for several clinically important associations. The separation of prevalence estimates by nutritional assessment tool avoids inappropriate pooling across conceptually different instruments. In addition, the assessment methods used in this paper, in addition to a simple overall quality score, incorporated judgments at the JBI and QUIPS domain levels, thereby improving transparency regarding sampling, outcome measurement, confounding factors, time bias, and statistical reporting. However, these judgments should be understood as structured descriptions of the limitations of the evidence, rather than proof of a generally low risk of bias in the included studies.

The limitations should also be recognized. First, all included studies were observational, and eight were cross-sectional, so the associations identified here cannot establish temporal sequence or causality. The QUIPS assessment further indicated that temporal ambiguity and incomplete confounding adjustment were important limitations for several associated-factor analyses, while the JBI assessment identified moderate concerns regarding sampling representativeness and response-rate reporting in some prevalence studies. Second, heterogeneity was substantial for prevalence estimates and for several associated factors, reflecting differences in cancer subsite, treatment phase, assessment timing, nutritional assessment tools, and variable definitions. Because most studies reported baseline or cross-sectional data, the pooled proportions should be interpreted as prevalence estimates rather than incidence estimates. Residual confounding is particularly relevant for dysphagia, hypoalbuminemia, low hemoglobin, tumor stage, and radiotherapy or chemoradiotherapy because these variables may reflect disease burden, inflammation, treatment toxicity, or intermediate mechanisms rather than independent etiological effects. Incorporation bias may also affect the association between low BMI and GLIM-defined malnutrition, because low BMI is part of the phenotypic diagnostic framework. In addition, prolonged tube feeding is particularly vulnerable to reverse causality and confounding by indication, as enteral support is often initiated after nutritional vulnerability has already become clinically apparent. Third, more than half of the included studies were conducted in China, which may limit generalizability to settings with different HNC epidemiology, dietary patterns, and treatment pathways. Fourth, several pooled estimates were based on only three or four studies, so the results should be considered clinically informative but still in need of confirmation. Fifth, many studies reported unadjusted or differently adjusted effect estimates, and individual patient data were not available for uniform adjustment. Finally, formal publication-bias testing was not conducted because no pooled outcome included at least 10 studies; small-study effects therefore cannot be fully excluded. In addition, residual statistical dependence can arise when reports share recruitment sites or participants, or when several assessment time points are drawn from the same cohort. This risk was addressed by the cohort-level audit and the one-estimate-per-cohort rule, but the definitive analyses require confirmation against the original extraction workbook.

### 4.6 Future directions

Future research should move beyond isolated associated-factor reporting toward longitudinal, standardized, and mechanism-informed designs (57). Prospective studies should collect repeated nutritional assessments before, during, and after treatment; use harmonized definitions for advanced age, low BMI, dysphagia, and treatment exposure; and report adjusted estimates that account for tumor stage, treatment modality, baseline nutritional status, inflammatory markers, and psychosocial variables. Individual patient data meta-analysis would allow more precise modeling of interactions among dysphagia, treatment intensity, inflammatory markers, and psychological distress.

Clinically, the next step is to develop and validate a practical HNC malnutritionprediction model using the associated variables summarized in this review. If externally validated, such a model could be linked to prespecified supportive-care actions, including dietitian referral, swallowing evaluation, symptom management, psychosocial assessment, and consideration of enteral nutrition. Randomized trials are needed to determine whether risk-stratified multidisciplinary care reduces severe weight loss, treatment interruptions, hospitalizations, or deterioration in quality of life.

## 5 Conclusion

This systematic review and meta-analysis demonstrates that malnutrition and nutritional risk affect a substantial proportion of patients with head and neck cancer, although the estimated prevalence differs according to the nutritional outcome construct and assessment instrument. GLIM-based estimates represent diagnostically defined malnutrition, PG-SGA estimates represent oncology-oriented nutritional assessment categories, and NRS 2002 estimates represent nutritional risk rather than definitive malnutrition diagnosis. Ten variables associated with malnutrition were identified and quantified, with psychosocial symptoms, functional impairment, advanced-stage disease, and treatment exposure showing the largest or most consistent associations. However, dysphagia, albumin, hemoglobin, tumor stage, and treatment modality should be interpreted as correlates and markers of disease burden, inflammation, or treatment toxicity rather than proven independent causal factors. These findings support comprehensive, risk-stratified nutritional screening and early multidisciplinary intervention in clinical practice. Integrating nutritional, swallowing, psychological, biochemical, and oncologic assessments at the time of HNC diagnosis may help identify patients who require earlier supportive care, although prospective studies are still needed to determine whether such risk-stratified interventions improve treatment tolerance, quality of life, or survival outcomes.

## Data Availability

The raw data supporting the conclusions of this article will be made available by the authors, without undue reservation.
