# Supplementary material for: Prevalence of malnutrition and nutritional risk, and associated factors in adults with head and neck cancer: a systematic review and meta-analysis
Source: Front Oncol. 2026 Aug 12;16:1905663. doi: 10.3389/fonc.2026.1905663 (PMC13506493; doi:10.3389/fonc.2026.1905663)
Supplement: Supplementary file 1 [file Table1.docx]

**Supplementary Table S1. Evidence Traceability and Extraction Details for the Meta-analysis Dataset.**

| **ID** | **Study / full citation identifier** | **DOI / PMID / database ID** | **Population and setting** | **Tool / cut-off / time point** | **Extracted effect estimate and reference category** | **Adjustment status and covariates** | **Exact meta-analysis contribution** | **Cohort-overlap audit decision** |
| --- | --- | --- | --- | --- | --- | --- | --- | --- |
| 1 | Hu H, Tu Y, Liang G, et al. Analysis of nutritional status and its influencing factors in patients with head and neck cancer: an observational study. Medicine. 2025;104(33):e43731. | DOI: 10.1097/MD.0000000000043731; PMID: 40826729 | Adult HNC patients at Zhejiang Cancer Hospital, China; perioperative observational longitudinal cohort; follow-up to 2 months after surgery; N=148. | NRS 2002; ≥3; perioperative/preoperative and follow-up assessments; one prespecified persistent-risk estimate retained. | Nutritional-risk prevalence: 38/148 (25.7%); associated-factor estimates extracted from the reported longitudinal/multivariable analyses. | Adjusted analyses; demographic, clinical, biochemical, nutritional, and treatment-related variables as specified in the source model. | Figure 2 and Table 2: NRS 2002 prevalence; associated-factor analyses where compatible. | Independent 148-patient report. Compared with Tu et al. (2024); different sample size, recruitment window, and analytic objective; retained. |
| 2 | Steer B, Loeliger J, Edbrooke L, Deftereos I, Laing E, Kiss N. Malnutrition prevalence according to the GLIM criteria in head and neck cancer patients undergoing cancer treatment. Nutrients. 2020;12(11):3493. | DOI: 10.3390/nu12113493; PMID: 33203000 | Adults with HNC receiving inpatient or ambulatory cancer treatment in Australia; cross-sectional program-based sample; N=217. | GLIM; ≥1 phenotypic and ≥1 etiologic criterion; assessment during cancer treatment. | GLIM-defined prevalence: 61/217 (28.1%); treatment-related evidence used only when extractable and non-duplicative. | Primary prevalence estimate unadjusted; validation/predictive analyses adjusted only as reported in the source. | Figure 2 and Table 2: GLIM prevalence; treatment-related subgroup evidence. | Independent Australian cohort; no matching site-period-author combination among other records; retained. |
| 3 | Einarsson S, Karlsson HE, Björ O, Haylock AK, Tiblom Ehrsson Y. Mapping impact factors leading to the GLIM diagnosis of malnutrition in patients with head and neck cancer. Clinical Nutrition ESPEN. 2020;40:149–155. | DOI: 10.1016/j.clnesp.2020.09.174; PMID: 33183529 | Hospital-based Swedish HNC treatment cohort with repeated nutritional follow-up; the independent prespecified assessment contributing to prevalence included N=57. | GLIM; phenotypic plus etiologic criteria; one prespecified time point used in the conventional prevalence meta-analysis. | GLIM-defined prevalence: 18/57 (31.6%); impact factors used only from the appropriate reported time point/model. | Models considered clinical stage, treatment, nutrition-impact symptoms, inflammatory/weight-related variables as reported. | Figure 2 and Table 2: GLIM prevalence; eligible associated-factor analyses. | Repeated assessments within the same author cohort were audited; only one prespecified estimate entered the conventional meta-analysis. |
| 4 | Orell HK, Pohju AK, Österlund P, Schwab US, Ravasco P, Mäkitie A. GLIM in diagnosing malnutrition and predicting outcome in ambulatory patients with head and neck cancer. Frontiers in Nutrition. 2022;9:1030619. | DOI: 10.3389/fnut.2022.1030619; PMID: 36483923 | Ambulatory adults with newly diagnosed HNC in Finland; prospective hospital-based cohort; N=65. | GLIM; phenotypic plus etiologic criteria; baseline/early-treatment assessment. | GLIM-defined prevalence: 24/65 (36.9%); prognostic estimates retained only for the source-defined outcome and time point. | Prevalence unadjusted; survival/prognostic models used source-specified demographic and clinical covariates. | Figure 2 and Table 2: GLIM prevalence; compatible prognostic/associated-factor evidence. | Independent Finnish ambulatory cohort; no apparent overlap; retained. |
| 5 | Wang P, Soh KL, Ying Y, et al. Risk factors for malnutrition in patients with nasopharyngeal carcinoma. Supportive Care in Cancer. 2023;31:723. | DOI: 10.1007/s00520-023-08166-8; PMID: 38008866 | NPC patients at the First Affiliated Hospital of Guangxi Medical University, China; September 2021–November 2022; cross-sectional; N=305. | GLIM; ≥1 phenotypic and ≥1 etiologic criterion; concurrent baseline/clinical assessment. | GLIM-defined prevalence: 143/305 (46.9%); adjusted ORs for source-defined malnutrition-associated variables. | Adjusted logistic model; BMI, radiation dose, appetite loss, prealbumin, and other candidate covariates reported by the authors. | Figure 2 and Table 2: GLIM prevalence; associated-factor meta-analyses where definitions matched. | Independent 305-patient Guangxi cohort. Corrected Figure 2 label/year and event count from 129 to 143; retained. |
| 6 | Zhan C, Gong Z, Ji N, Ju X. Risk factors of malnutrition in nasopharyngeal carcinoma patients undergoing radiotherapy and construction of a predictive model. Journal of Nutritional Science and Vitaminology. 2025;71(6):537. | DOI: 10.3177/jnsv.71.537; PMID: 41485969 | NPC patients undergoing radiotherapy at the Second Affiliated Hospital of Hainan Medical University, China; March 2023–November 2024; retrospective; N=158. | PG-SGA; source-defined moderate/severe malnutrition; radiotherapy assessment. | PG-SGA-defined prevalence: 118/158 (74.7%); prediction-model factor estimates considered separately from the prevalence analysis. | Multivariable/prediction model using source-specified demographic, clinical, laboratory, and treatment variables. | Figure 2 and Table 2: PG-SGA prevalence; model/associated-factor analyses where extractable. | Independent Hainan cohort; no matching site-period cohort identified; retained. |
| 7 | Taouchikht M, Fares H, Abdou S, et al. Evaluating nutritional health in cases of head and neck cancer receiving radiotherapy. International Journal of Innovative Science and Research Technology. 2024:2724–2730. | DOI: 10.38124/ijisrt/IJISRT24AUG437 | Adults with HNC receiving radiotherapy at the National Institute of Oncology, Rabat, Morocco; October 2023–March 2024; prospective observational; N=31. | PG-SGA; categories B/C; assessment at the end of radiotherapy. | PG-SGA-defined prevalence: 24/31 (77.4%); descriptive treatment-course nutritional changes. | Mainly unadjusted descriptive/longitudinal comparisons; no adjusted effect entered in factor meta-analysis unless explicitly reported. | Figure 2 and Table 2: PG-SGA prevalence. | Independent Moroccan cohort; Figure 2 event count corrected from 25 to source-reported 24; retained. |
| 8 | Lima ENS, et al. Associations of nutritional risk, pre-treatment malnutrition, and cachexia with clinical outcomes in patients with head and neck cancer. BMC Cancer. 2026. | DOI: 10.1186/s12885-025-15373-5 | Adults with HNSCC at a Brazilian tertiary reference outpatient clinic; baseline July 2017–November 2018 and follow-up to November 2023; prospective; N=84. | PG-SGA; categories B/C; pretreatment baseline assessment. | PG-SGA-defined prevalence: 52/84 (61.9%); source-reported associations with mortality, recurrence, and metastasis. | Multivariable outcome models using source-specified clinical, nutritional, disease, and treatment covariates. | Figure 2 and Table 2: PG-SGA prevalence; eligible prognostic/associated-factor analyses. | Independent Brazilian cohort; event count unchanged at 52/84; retained. |
| 9 | Noronha V, Chawda A, et al. Nutritional status and impact on outcomes of patients with locally advanced head and neck squamous cell carcinoma treated with concurrent chemoradiotherapy: a pre-planned secondary analysis of a phase 3 randomized controlled trial. Journal of the Egyptian National Cancer Institute. 2025;37(1). | DOI: 10.1186/s43046-025-00305-y; PMID: 40717193 | Patients with laryngeal/hypopharyngeal LAHNSCC at Tata Memorial Hospital, India; phase III trial cohort recruited 2013–2017; secondary analysis; N=112. | PG-SGA; score ≥4; baseline assessment selected for prevalence; subsequent CRT assessments treated as correlated. | Baseline PG-SGA-defined prevalence: 64/112 (57.1%); outcome associations extracted from the prespecified secondary analysis. | Trial/secondary-analysis models used source-specified disease, treatment-arm, clinical, and nutritional variables. | Figure 2 and Table 2: PG-SGA prevalence; eligible treatment/outcome associations. | Multiple time points from the same trial cohort were audited; one baseline estimate retained for conventional meta-analysis. |
| 10 | Deng LH, Chi K, Zong Y, Li Y, Chen M, Chen P. Malnutrition in patients with head and neck cancer undergoing radiotherapy: a cross-sectional study. European Journal of Oncology Nursing. 2023;66:102387. | DOI: 10.1016/j.ejon.2023.102387; PMID: 37611500 | Consecutive HNC patients receiving radiotherapy at Qingdao Municipal Hospital, China; January 2019–June 2020; cross-sectional; N=108. | NRS 2002; ≥3; assessment during radiotherapy. | Nutritional-risk prevalence: 52/108 (48.1%); source-reported associations for age, stage, radiation site, diabetes, anxiety/depression. | Univariable and source-reported multivariable analyses; clinical, disease, metabolic, and psychological covariates. | Figure 2 and Table 2: NRS 2002 prevalence; compatible associated-factor analyses. | Independent Qingdao cohort; no apparent overlap; retained. |
| 11 | Wang P, Huang X, Liu Y, et al. Risk factors and the nomogram model for malnutrition in patients with nasopharyngeal carcinoma. Supportive Care in Cancer. 2024;32:256. | DOI: 10.1007/s00520-024-08459-6 | Chinese NPC malnutrition nomogram development cohort; baseline/model assessment; N=383. | Source-defined malnutrition outcome; baseline model assessment; definition retained exactly as reported by the source. | Model outcome: 116/383 (30.3%); adjusted prediction-model coefficients/ORs and reference categories as reported. | Adjusted multivariable nomogram; candidate clinical, nutritional, laboratory, and treatment covariates specified in the source. | Associated-factor/model analyses only; excluded from Figure 2 and construct-specific prevalence pooling. | Compared with Zhu et al. (2025): different article, recruitment framework, outcome definition, and analysis; not treated as a duplicate; not plotted. |
| 12 | Zhu B, et al. A dynamic online nomogram for predicting nutritional risk in nasopharyngeal carcinoma patients after radiotherapy. Supportive Care in Cancer. 2025. | DOI: 10.1007/s00520-025-09547-x; PMID: 40434547 | NPC patients after radiotherapy at Nanfang and Zhujiang Hospitals, China; January 2021–January 2023; two-center retrospective cohort; N=383. | NRS 2002; source-reported threshold; post-radiotherapy assessment. | Nutritional-risk prevalence: 116/383 (30.3%); prediction-model effects extracted with source reference categories. | Multivariable/dynamic nomogram using source-specified clinical, treatment, symptom, and laboratory predictors. | Figure 2 and Table 2: NRS 2002 prevalence; eligible model/associated-factor evidence. | Sites, recruitment dates, author group, and analysis differ from Wang et al. (2024); treated as an independent cohort. Figure count corrected from 51 to 116. |
| 13 | Wang G, Ou M, Chen H, Zhu S, Chen Y, Xu X. Perioperative nutritional risk and its influencing factors in patients with oral cancer: a longitudinal study. Frontiers in Nutrition. 2023;10:1200820. | DOI: 10.3389/fnut.2023.1200820; PMID: 37426177 | Perioperative oral-cancer patients in China; longitudinal hospital cohort; N=198. | NRS 2002; ≥3; three perioperative time points; the prespecified postoperative/perioperative estimate 104/198 was used. | Nutritional-risk prevalence: 104/198 (52.5%); longitudinal coefficients for education, smoking, stage, flap repair, tracheotomy, and other reported factors. | Generalized estimating equation with source-specified demographic, clinical, surgical, and treatment variables. | Figure 2 and Table 2: NRS 2002 prevalence; eligible associated-factor analyses. | Correlated time points were not entered simultaneously; one prespecified estimate retained. |
| 14 | Chen XY, Li CL, Qin XJ, Zhang JF. Factors influencing nutrition trajectories of patients with oral cancer. China Journal of Oral and Maxillofacial Surgery. 2023;21(6):538–547. | DOI: 10.19438/j.cjoms.2023.06.002; Chinese database record | Oral-cancer patients at Shanghai Ninth People's Hospital, China; followed from preoperative assessment to 3 months postoperatively; longitudinal; N=269. | PG-SGA; source-defined malnutrition trajectory/category; one prespecified estimate retained. | PG-SGA-defined prevalence: 46/269 (17.1%); trajectory-class predictors extracted using the source reference categories. | Trajectory/model analyses using source-specified sociodemographic, clinical, surgical, and treatment covariates. | Figure 2 and Table 2: PG-SGA prevalence; eligible trajectory-associated factors. | Repeated measurements were audited; one prespecified estimate entered the conventional prevalence meta-analysis. |
| 15 | Liu M, Gao F, An R, Wu Z, Chen W. Correlation analysis between prognostic nutritional index trajectory categories and radiotherapy-induced severe oral mucositis in head and neck cancer patients undergoing radiotherapy. Oral Health & Preventive Dentistry. 2024;22:671–680. | DOI: 10.3290/j.ohpd.b5877400; PMID: 39665402 | HNC/NPC patients receiving radiotherapy in Nanning, China; observational cohort; N=281. | Prognostic Nutritional Index trajectory/combined nutritional index; serial radiotherapy assessments. | Source-defined adverse nutritional/PNI trajectory: 137/281 (48.8%); trajectory association with severe radiation-induced oral mucositis. | Source-reported trajectory and multivariable analyses with clinical, treatment, inflammatory, and nutritional variables. | Associated-factor analysis only; different nutritional construct and therefore excluded from Figure 2 prevalence pooling. | Distinct outcome construct and cohort; retained for associated-factor evidence only. |
| 16 | Tu Y, Chen F, Yu Q, Song L, Chen M. Application of NRS2002 and PG-SGA in nutritional assessment for perioperative patients with head and neck squamous cell carcinoma: an observational study. Medicine. 2024;103:e40025. | DOI: 10.1097/MD.0000000000040025; PMID: 39470500 | Perioperative HNSCC patients at Zhejiang Cancer Hospital, China; January 2017–December 2020; observational comparative cohort; N=209. | NRS 2002; ≥3; day-of-admission estimate used. PG-SGA was evaluated concurrently but not added as a correlated second estimate to the same pooled analysis. | Admission NRS 2002 prevalence: 18/209 (8.6%); comparative diagnostic/assessment results reported by the study. | Primarily instrument-comparison and descriptive analyses; adjusted effects used only if clearly reported. | Figure 2 and Table 2: NRS 2002 prevalence. | Independent 209-patient cohort; replaces the erroneous duplicated 38/148 entry. Compared with Hu et al. (2025) and retained as distinct. |
| 17 | Wang P, Huang X, Xue L, et al. Nutritional risk factors in patients with nasopharyngeal carcinoma: a cross-sectional study. Frontiers in Nutrition. 2024;11:1386361. | DOI: 10.3389/fnut.2024.1386361 | NPC patients in a Guangxi clinical cohort, China; January 2022–May 2023; prospective cross-sectional; N=377. | NRS 2002; ≥3; admission/baseline assessment. | Nutritional-risk prevalence: 155/377 (41.1%); source-reported adjusted associations with nutritional risk. | Adjusted logistic analysis using source-specified demographic, clinical, treatment, symptom, and laboratory covariates. | Figure 2 and Table 2: NRS 2002 prevalence; eligible associated-factor analyses. | Compared with other Guangxi/Wang records by site, dates, sample size, author group, and outcome; treated as an independent cohort. |
| 18 | Vílchez-López FJ, González-Pacheco M, Fernández-Jiménez R, et al. Predictive factors of the degrees of malnutrition according to GLIM criteria in head and neck cancer patients: VALOR Group. Cancers. 2024;16(24):4255. | DOI: 10.3390/cancers16244255; PMID: 39766154 | Multicenter VALOR Group cohort of HNC patients in Spain; morphofunctional assessment during the first 2 weeks of radiotherapy; N=514. | GLIM criteria; early-radiotherapy assessment; malnutrition degree classified using source-defined GLIM rules. | GLIM-defined prevalence: 265/514 (51.6%); multivariable predictive factors and morphofunctional associations. | Multivariable logistic/random-forest analyses with clinical, biochemical, functional, body-composition, and treatment variables. | Figure 2 and Table 2: GLIM prevalence; eligible associated-factor analyses. | Independent multicenter Spanish cohort; no apparent overlap with other records; retained. |
| 19 | Kuang L, Yu J, Zhou Y, et al. Predicting postoperative malnutrition in patients with oral cancer: development of an XGBoost model with SHAP analysis and web-based application. Frontiers in Oncology. 2025;15:1564459. | DOI: 10.3389/fonc.2025.1564459; PMID: 40421091 | Postoperative oral-cancer patients at a tertiary hospital in China; August 2023–May 2024; prospective model-development cohort; N=487. | GLIM-defined postoperative malnutrition; postoperative model outcome. | Postoperative GLIM outcome: 251/487 (51.5%); machine-learning feature effects/importance extracted as model evidence rather than conventional adjusted ORs. | XGBoost and comparator models with SHAP interpretation; source-specified clinical, laboratory, and perioperative predictors. | Associated-factor/model analyses only; postoperative construct excluded from Figure 2 prevalence pooling. | Independent postoperative oral-cancer cohort; retained for model evidence only. |
| 20 | Tsai YT, Lai CH, Huang TH, et al. Association of malnutrition with postoperative complication risk after curative surgery for oral cancer: observational study. Medicine. 2020;99(52):e23860. | DOI: 10.1097/MD.0000000000023860; PMID: 33350779 | Patients undergoing curative surgery for oral squamous cell carcinoma at a single institution in Taiwan, China; January 2014–December 2018; retrospective; N=70. | PG-SGA; categories B/C; preoperative assessment. | PG-SGA-defined prevalence: 44/70 (62.9%); postoperative-complication association with source reference categories. | Source-reported regression/clinical analyses considering nutritional status and perioperative clinical covariates. | Figure 2 and Table 2: PG-SGA prevalence; eligible postoperative-complication association. | Independent 70-patient OSCC cohort. Corrects the erroneous ‘Fukumoto et al.’ label; retained. |

*Note. Each conventional prevalence meta-analysis included one prespecified estimate per independent cohort. Multiple time points or instrument results from the same participants were not entered as independent observations. The 17 prevalence estimates map one-to-one to Figure 2; three additional studies (IDs 11, 15, and 19) contributed only to associated-factor or model analyses. HNC, head and neck cancer; NPC, nasopharyngeal carcinoma; OSCC, oral squamous cell carcinoma; GLIM, Global Leadership Initiative on Malnutrition; PG-SGA, Patient-Generated Subjective Global Assessment; NRS 2002, Nutritional Risk Screening 2002; PNI, Prognostic Nutritional Index.*

**Supplementary Table S2. Database-specific search strategies, platforms, search dates, and record counts**

| **Database** | **Platform** | **Search date** | **Search field and strategy** | **Records before deduplication** | **Unique records retained after cross-database deduplication** |
| --- | --- | --- | --- | --- | --- |
| PubMed/MEDLINE | NCBI | 4 July 2026 | ("Head and Neck Neoplasms"[Mesh] OR "head and neck cancer"[tiab] OR "oral cancer"[tiab] OR "nasopharyngeal carcinoma"[tiab] OR "laryngeal cancer"[tiab] OR "oropharyngeal cancer"[tiab] OR "hypopharyngeal cancer"[tiab]) AND ("Malnutrition"[Mesh] OR malnutrition[tiab] OR "nutritional status"[tiab] OR "nutritional risk"[tiab] OR undernutrition[tiab] OR cachexia[tiab] OR "nutritional screening"[tiab] OR "nutritional assessment"[tiab]) AND ("risk factor*"[tiab] OR predictor*[tiab] OR determinant*[tiab] OR associated[tiab] OR prevalence[tiab] OR incidence[tiab]) | 812 | 812 |
| Embase | Elsevier | 4 July 2026 | ('head and neck tumor'/exp OR 'head and neck cancer':ti,ab OR 'oral cancer':ti,ab OR 'nasopharyngeal carcinoma':ti,ab OR 'laryngeal cancer':ti,ab OR 'oropharyngeal cancer':ti,ab OR 'hypopharyngeal cancer':ti,ab) AND ('malnutrition'/exp OR malnutrition:ti,ab OR 'nutritional status':ti,ab OR 'nutritional risk':ti,ab OR undernutrition:ti,ab OR cachexia:ti,ab) AND ('risk factor'/exp OR predictor*:ti,ab OR determinant*:ti,ab OR associated:ti,ab OR prevalence:ti,ab OR incidence:ti,ab) | 1,264 | 902 |
| Web of Science Core Collection | Clarivate | 4 July 2026 | TS=("head and neck cancer" OR "head and neck neoplasm*" OR "oral cancer" OR "nasopharyngeal carcinoma" OR "laryngeal cancer" OR "oropharyngeal cancer" OR "hypopharyngeal cancer") AND TS=(malnutrition OR "nutritional status" OR "nutritional risk" OR undernutrition OR cachexia OR "nutritional screening" OR "nutritional assessment") AND TS=("risk factor*" OR predictor* OR determinant* OR associated OR prevalence OR incidence) | 1,038 | 704 |
| Cochrane Library | Wiley | 4 July 2026 | Title Abstract Keyword: ("head and neck cancer" OR "head and neck neoplasms" OR "oral cancer" OR "nasopharyngeal carcinoma" OR "laryngeal cancer") AND (malnutrition OR "nutritional status" OR "nutritional risk" OR cachexia) AND ("risk factors" OR predictors OR prevalence OR incidence) | 164 | 113 |
| CINAHL | EBSCOhost | 4 July 2026 | (MH "Head and Neck Neoplasms+" OR TI/AB "head and neck cancer" OR TI/AB "oral cancer" OR TI/AB "nasopharyngeal carcinoma") AND (MH "Malnutrition+" OR TI/AB malnutrition OR TI/AB "nutritional status" OR TI/AB "nutritional risk") AND (MH "Risk Factors+" OR TI/AB predictor* OR TI/AB associated OR TI/AB prevalence) | 426 | 287 |
| CNKI | CNKI | 4 July 2026 | 主题=(头颈癌 OR 头颈部肿瘤 OR 口腔癌 OR 鼻咽癌 OR 喉癌 OR 下咽癌) AND 主题=(营养不良 OR 营养风险 OR 营养状态 OR 营养筛查 OR 恶病质) AND 主题=(危险因素 OR 影响因素 OR 相关因素 OR 预测因素 OR 发生率 OR 患病率) | 799 | 626 |
| Wanfang | Wanfang Data | 4 July 2026 | 主题或摘要=(头颈癌 OR 头颈部肿瘤 OR 口腔癌 OR 鼻咽癌 OR 喉癌 OR 下咽癌) AND (营养不良 OR 营养风险 OR 营养状态 OR 营养评估) AND (危险因素 OR 影响因素 OR 相关因素 OR 预测因素) | 713 | 437 |
| VIP | VIP Database | 4 July 2026 | 题名/关键词/摘要=(头颈癌 OR 头颈部肿瘤 OR 口腔癌 OR 鼻咽癌 OR 喉癌) AND (营养不良 OR 营养风险 OR 营养状态 OR 营养筛查) AND (危险因素 OR 影响因素 OR 相关因素 OR 预测因素) | 536 | 322 |
| CBM/SinoMed | CBM | 4 July 2026 | 主题词/自由词=(头颈部肿瘤 OR 口腔肿瘤 OR 鼻咽肿瘤 OR 喉肿瘤 OR 下咽肿瘤) AND (营养不良 OR 营养风险 OR 营养状况 OR 营养评价) AND (危险因素 OR 影响因素 OR 相关因素 OR 预测因素 OR 患病率 OR 发生率) | 591 | 451 |
| Citation searching and guideline sources | Manual search | 4 July 2026 | Reference lists of included studies and relevant reviews; ESPEN, ASPEN, ASCO, ESMO, and head-and-neck oncology guideline repositories | 96 | 73 |
| **Total** | **-** | **-** | **-** | **6,439** | **4,727** |

**Supplementary Table S3. Domain-level risk-of-bias judgments for included studies**

| **ID** | **Study** | **Design / outcome contribution** | **JBI: sampling frame & recruitment** | **JBI: sample description & size** | **JBI: outcome definition & measurement** | **JBI: response / completeness / analysis** | **QUIPS: participation / attrition** | **QUIPS: factor & outcome measurement** | **QUIPS: confounding control** | **QUIPS: statistical analysis / reporting** | **Overall judgment and main rationale** |
| --- | --- | --- | --- | --- | --- | --- | --- | --- | --- | --- | --- |
| 1 | Hu et al. (2025) | Observational longitudinal; NRS 2002 prevalence and associated-factor estimates | L/M: multicenter or clearly defined consecutive/clinical cohort; recruitment frame is relevant to the target HNC population. | L: participant characteristics, setting, sample size, and analysis denominator are adequately reported. | L: the nutritional instrument and source-defined threshold/time point are explicit and applied consistently. | L/M: the analysis denominator is clear; missingness/response handling is adequate or unlikely to materially alter the estimate. | L/M: cohort entry and analysis population are sufficiently described; attrition is considered for repeated assessments. | L: nutritional outcome and major clinical factors are measured with established tools or clearly defined source variables. | M: adjusted model is reported with clinically relevant covariates; residual and unmeasured confounding remain possible. | M: analysis is appropriate to the design; precision, multiplicity, or small-event reporting is incomplete in places. | Low-to-moderate concern: outcome ascertainment and traceability are strong; remaining concerns relate mainly to observational design and residual confounding. |
| 2 | Steer et al. (2020) | Cross-sectional; GLIM prevalence | M: single-center or convenience/clinical sampling; recruitment is described but representativeness is limited. | M: sample size and core characteristics are reported; some setting, missing-data, or precision details are incomplete. | L: the nutritional instrument and source-defined threshold/time point are explicit and applied consistently. | M: completeness and response handling are not fully detailed; complete-case or small-sample bias is possible. | M: participation is adequately described, although cross-sectional/convenience selection may limit representativeness. | L/M: the principal outcome is measured using a recognized tool; some factor measurement details are limited. | M/H: prevalence-focused or mainly unadjusted analysis; confounding control is limited for association claims. | M: analysis is appropriate to the design; precision, multiplicity, or small-event reporting is incomplete in places. | Moderate concern: the study is usable for its prespecified contribution, with limitations from single-center/small-sample design, incomplete adjustment, or response reporting. |
| 3 | Einarsson et al. (2020) | Prospective observational/longitudinal; GLIM prevalence and impact factors | M: single-center or convenience/clinical sampling; recruitment is described but representativeness is limited. | M: sample size and core characteristics are reported; some setting, missing-data, or precision details are incomplete. | L: the nutritional instrument and source-defined threshold/time point are explicit and applied consistently. | M: completeness and response handling are not fully detailed; complete-case or small-sample bias is possible. | L/M: cohort entry and analysis population are sufficiently described; attrition is considered for repeated assessments. | L/M: the principal outcome is measured using a recognized tool; some factor measurement details are limited. | M: adjusted model is reported with clinically relevant covariates; residual and unmeasured confounding remain possible. | M: analysis is appropriate to the design; precision, multiplicity, or small-event reporting is incomplete in places. | Moderate concern: the study is usable for its prespecified contribution, with limitations from single-center/small-sample design, incomplete adjustment, or response reporting. |
| 4 | Orell et al. (2022) | Prospective cohort; GLIM prevalence and prognostic evidence | L/M: multicenter or clearly defined consecutive/clinical cohort; recruitment frame is relevant to the target HNC population. | L: participant characteristics, setting, sample size, and analysis denominator are adequately reported. | L: the nutritional instrument and source-defined threshold/time point are explicit and applied consistently. | L/M: the analysis denominator is clear; missingness/response handling is adequate or unlikely to materially alter the estimate. | L/M: cohort entry and analysis population are sufficiently described; attrition is considered for repeated assessments. | L: nutritional outcome and major clinical factors are measured with established tools or clearly defined source variables. | M/H: prevalence-focused or mainly unadjusted analysis; confounding control is limited for association claims. | M: analysis is appropriate to the design; precision, multiplicity, or small-event reporting is incomplete in places. | Low-to-moderate concern: outcome ascertainment and traceability are strong; remaining concerns relate mainly to observational design and residual confounding. |
| 5 | Wang et al. (2023) | Cross-sectional; GLIM prevalence and associated factors | L/M: multicenter or clearly defined consecutive/clinical cohort; recruitment frame is relevant to the target HNC population. | L: participant characteristics, setting, sample size, and analysis denominator are adequately reported. | L: the nutritional instrument and source-defined threshold/time point are explicit and applied consistently. | L/M: the analysis denominator is clear; missingness/response handling is adequate or unlikely to materially alter the estimate. | M: participation is adequately described, although cross-sectional/convenience selection may limit representativeness. | L: nutritional outcome and major clinical factors are measured with established tools or clearly defined source variables. | M: adjusted model is reported with clinically relevant covariates; residual and unmeasured confounding remain possible. | M: analysis is appropriate to the design; precision, multiplicity, or small-event reporting is incomplete in places. | Low-to-moderate concern: outcome ascertainment and traceability are strong; remaining concerns relate mainly to observational design and residual confounding. |
| 6 | Zhan et al. (2025) | Retrospective model-development; PG-SGA prevalence and predictors | M: single-center or convenience/clinical sampling; recruitment is described but representativeness is limited. | M: sample size and core characteristics are reported; some setting, missing-data, or precision details are incomplete. | L: the nutritional instrument and source-defined threshold/time point are explicit and applied consistently. | M: completeness and response handling are not fully detailed; complete-case or small-sample bias is possible. | M: participation is adequately described, although cross-sectional/convenience selection may limit representativeness. | L/M: the principal outcome is measured using a recognized tool; some factor measurement details are limited. | M: adjusted model is reported with clinically relevant covariates; residual and unmeasured confounding remain possible. | L/M: model specification and principal estimates are reported; internal validation/diagnostics are available where applicable. | Moderate concern: the study is usable for its prespecified contribution, with limitations from single-center/small-sample design, incomplete adjustment, or response reporting. |
| 7 | Taouchikht et al. (2024) | Prospective observational; PG-SGA prevalence | M: single-center or convenience/clinical sampling; recruitment is described but representativeness is limited. | M: sample size and core characteristics are reported; some setting, missing-data, or precision details are incomplete. | M: a recognized instrument is used, but operational detail or timing is incompletely reported. | M: completeness and response handling are not fully detailed; complete-case or small-sample bias is possible. | L/M: cohort entry and analysis population are sufficiently described; attrition is considered for repeated assessments. | L/M: the principal outcome is measured using a recognized tool; some factor measurement details are limited. | M/H: prevalence-focused or mainly unadjusted analysis; confounding control is limited for association claims. | M: analysis is appropriate to the design; precision, multiplicity, or small-event reporting is incomplete in places. | Moderate concern: the study is usable for its prespecified contribution, with limitations from single-center/small-sample design, incomplete adjustment, or response reporting. |
| 8 | Lima et al. (2026) | Prospective cohort; PG-SGA prevalence and prognostic factors | L/M: multicenter or clearly defined consecutive/clinical cohort; recruitment frame is relevant to the target HNC population. | L: participant characteristics, setting, sample size, and analysis denominator are adequately reported. | L: the nutritional instrument and source-defined threshold/time point are explicit and applied consistently. | L/M: the analysis denominator is clear; missingness/response handling is adequate or unlikely to materially alter the estimate. | L/M: cohort entry and analysis population are sufficiently described; attrition is considered for repeated assessments. | L: nutritional outcome and major clinical factors are measured with established tools or clearly defined source variables. | M: adjusted model is reported with clinically relevant covariates; residual and unmeasured confounding remain possible. | M: analysis is appropriate to the design; precision, multiplicity, or small-event reporting is incomplete in places. | Low-to-moderate concern: outcome ascertainment and traceability are strong; remaining concerns relate mainly to observational design and residual confounding. |
| 9 | Noronha et al. (2025) | Pre-planned secondary RCT analysis; PG-SGA prevalence and outcomes | L/M: multicenter or clearly defined consecutive/clinical cohort; recruitment frame is relevant to the target HNC population. | L: participant characteristics, setting, sample size, and analysis denominator are adequately reported. | L: the nutritional instrument and source-defined threshold/time point are explicit and applied consistently. | L/M: the analysis denominator is clear; missingness/response handling is adequate or unlikely to materially alter the estimate. | M: participation is adequately described, although cross-sectional/convenience selection may limit representativeness. | L: nutritional outcome and major clinical factors are measured with established tools or clearly defined source variables. | M: adjusted model is reported with clinically relevant covariates; residual and unmeasured confounding remain possible. | M: analysis is appropriate to the design; precision, multiplicity, or small-event reporting is incomplete in places. | Low-to-moderate concern: outcome ascertainment and traceability are strong; remaining concerns relate mainly to observational design and residual confounding. |
| 10 | Deng et al. (2023) | Cross-sectional; NRS 2002 prevalence and associated factors | M: single-center or convenience/clinical sampling; recruitment is described but representativeness is limited. | M: sample size and core characteristics are reported; some setting, missing-data, or precision details are incomplete. | L: the nutritional instrument and source-defined threshold/time point are explicit and applied consistently. | M: completeness and response handling are not fully detailed; complete-case or small-sample bias is possible. | M: participation is adequately described, although cross-sectional/convenience selection may limit representativeness. | L/M: the principal outcome is measured using a recognized tool; some factor measurement details are limited. | M: adjusted model is reported with clinically relevant covariates; residual and unmeasured confounding remain possible. | M: analysis is appropriate to the design; precision, multiplicity, or small-event reporting is incomplete in places. | Moderate concern: the study is usable for its prespecified contribution, with limitations from single-center/small-sample design, incomplete adjustment, or response reporting. |
| 11 | Wang et al. (2024; nomogram) | Model-development; source-defined malnutrition and associated factors | M: single-center or convenience/clinical sampling; recruitment is described but representativeness is limited. | M: sample size and core characteristics are reported; some setting, missing-data, or precision details are incomplete. | L/M: the source-defined model/trajectory outcome is reproducible, but it differs from the three primary prevalence constructs. | M: completeness and response handling are not fully detailed; complete-case or small-sample bias is possible. | M: participation is adequately described, although cross-sectional/convenience selection may limit representativeness. | L/M: the principal outcome is measured using a recognized tool; some factor measurement details are limited. | M: adjusted model is reported with clinically relevant covariates; residual and unmeasured confounding remain possible. | L/M: model specification and principal estimates are reported; internal validation/diagnostics are available where applicable. | Moderate concern: the study is usable for its prespecified contribution, with limitations from single-center/small-sample design, incomplete adjustment, or response reporting. |
| 12 | Zhu et al. (2025) | Two-center retrospective model study; NRS 2002 prevalence and predictors | L/M: multicenter or clearly defined consecutive/clinical cohort; recruitment frame is relevant to the target HNC population. | L: participant characteristics, setting, sample size, and analysis denominator are adequately reported. | L: the nutritional instrument and source-defined threshold/time point are explicit and applied consistently. | L/M: the analysis denominator is clear; missingness/response handling is adequate or unlikely to materially alter the estimate. | M: participation is adequately described, although cross-sectional/convenience selection may limit representativeness. | L: nutritional outcome and major clinical factors are measured with established tools or clearly defined source variables. | M: adjusted model is reported with clinically relevant covariates; residual and unmeasured confounding remain possible. | L/M: model specification and principal estimates are reported; internal validation/diagnostics are available where applicable. | Low-to-moderate concern: outcome ascertainment and traceability are strong; remaining concerns relate mainly to observational design and residual confounding. |
| 13 | Wang et al. (2023; oral cancer) | Longitudinal; NRS 2002 prevalence and influencing factors | L/M: multicenter or clearly defined consecutive/clinical cohort; recruitment frame is relevant to the target HNC population. | L: participant characteristics, setting, sample size, and analysis denominator are adequately reported. | L: the nutritional instrument and source-defined threshold/time point are explicit and applied consistently. | L/M: the analysis denominator is clear; missingness/response handling is adequate or unlikely to materially alter the estimate. | L/M: cohort entry and analysis population are sufficiently described; attrition is considered for repeated assessments. | L: nutritional outcome and major clinical factors are measured with established tools or clearly defined source variables. | M: adjusted model is reported with clinically relevant covariates; residual and unmeasured confounding remain possible. | M: analysis is appropriate to the design; precision, multiplicity, or small-event reporting is incomplete in places. | Low-to-moderate concern: outcome ascertainment and traceability are strong; remaining concerns relate mainly to observational design and residual confounding. |
| 14 | Chen et al. (2023) | Longitudinal trajectory study; PG-SGA prevalence and associated factors | M: single-center or convenience/clinical sampling; recruitment is described but representativeness is limited. | M: sample size and core characteristics are reported; some setting, missing-data, or precision details are incomplete. | L: the nutritional instrument and source-defined threshold/time point are explicit and applied consistently. | M: completeness and response handling are not fully detailed; complete-case or small-sample bias is possible. | L/M: cohort entry and analysis population are sufficiently described; attrition is considered for repeated assessments. | L/M: the principal outcome is measured using a recognized tool; some factor measurement details are limited. | M: adjusted model is reported with clinically relevant covariates; residual and unmeasured confounding remain possible. | M: analysis is appropriate to the design; precision, multiplicity, or small-event reporting is incomplete in places. | Moderate concern: the study is usable for its prespecified contribution, with limitations from single-center/small-sample design, incomplete adjustment, or response reporting. |
| 15 | Liu et al. (2024) | Observational trajectory study; PNI and oral-mucositis association | M: single-center or convenience/clinical sampling; recruitment is described but representativeness is limited. | M: sample size and core characteristics are reported; some setting, missing-data, or precision details are incomplete. | L/M: the source-defined model/trajectory outcome is reproducible, but it differs from the three primary prevalence constructs. | M: completeness and response handling are not fully detailed; complete-case or small-sample bias is possible. | M: participation is adequately described, although cross-sectional/convenience selection may limit representativeness. | L/M: the principal outcome is measured using a recognized tool; some factor measurement details are limited. | M: adjusted model is reported with clinically relevant covariates; residual and unmeasured confounding remain possible. | M: analysis is appropriate to the design; precision, multiplicity, or small-event reporting is incomplete in places. | Moderate concern: the study is usable for its prespecified contribution, with limitations from single-center/small-sample design, incomplete adjustment, or response reporting. |
| 16 | Tu et al. (2024) | Observational comparative; NRS 2002 prevalence and tool agreement | L/M: multicenter or clearly defined consecutive/clinical cohort; recruitment frame is relevant to the target HNC population. | L: participant characteristics, setting, sample size, and analysis denominator are adequately reported. | L: the nutritional instrument and source-defined threshold/time point are explicit and applied consistently. | L/M: the analysis denominator is clear; missingness/response handling is adequate or unlikely to materially alter the estimate. | M: participation is adequately described, although cross-sectional/convenience selection may limit representativeness. | L: nutritional outcome and major clinical factors are measured with established tools or clearly defined source variables. | M/H: prevalence-focused or mainly unadjusted analysis; confounding control is limited for association claims. | M: analysis is appropriate to the design; precision, multiplicity, or small-event reporting is incomplete in places. | Low-to-moderate concern: outcome ascertainment and traceability are strong; remaining concerns relate mainly to observational design and residual confounding. |
| 17 | Wang et al. (2024; nutritional risk) | Prospective cross-sectional; NRS 2002 prevalence and risk factors | L/M: multicenter or clearly defined consecutive/clinical cohort; recruitment frame is relevant to the target HNC population. | L: participant characteristics, setting, sample size, and analysis denominator are adequately reported. | L: the nutritional instrument and source-defined threshold/time point are explicit and applied consistently. | L/M: the analysis denominator is clear; missingness/response handling is adequate or unlikely to materially alter the estimate. | L/M: cohort entry and analysis population are sufficiently described; attrition is considered for repeated assessments. | L: nutritional outcome and major clinical factors are measured with established tools or clearly defined source variables. | M: adjusted model is reported with clinically relevant covariates; residual and unmeasured confounding remain possible. | M: analysis is appropriate to the design; precision, multiplicity, or small-event reporting is incomplete in places. | Low-to-moderate concern: outcome ascertainment and traceability are strong; remaining concerns relate mainly to observational design and residual confounding. |
| 18 | Vílchez-López et al. (2024) | Multicenter observational; GLIM prevalence and predictive factors | L: multicenter clinical sampling with clearly defined HNC eligibility and recruitment setting. | L: participant characteristics, setting, sample size, and analysis denominator are adequately reported. | L: the nutritional instrument and source-defined threshold/time point are explicit and applied consistently. | L/M: the analysis denominator is clear; missingness/response handling is adequate or unlikely to materially alter the estimate. | M: participation is adequately described, although cross-sectional/convenience selection may limit representativeness. | L: nutritional outcome and major clinical factors are measured with established tools or clearly defined source variables. | M: adjusted model is reported with clinically relevant covariates; residual and unmeasured confounding remain possible. | L/M: model specification and principal estimates are reported; internal validation/diagnostics are available where applicable. | Low-to-moderate concern: outcome ascertainment and traceability are strong; remaining concerns relate mainly to observational design and residual confounding. |
| 19 | Kuang et al. (2025) | Prospective model-development; postoperative GLIM outcome | L/M: multicenter or clearly defined consecutive/clinical cohort; recruitment frame is relevant to the target HNC population. | L: participant characteristics, setting, sample size, and analysis denominator are adequately reported. | L/M: the source-defined model/trajectory outcome is reproducible, but it differs from the three primary prevalence constructs. | L/M: the analysis denominator is clear; missingness/response handling is adequate or unlikely to materially alter the estimate. | L/M: cohort entry and analysis population are sufficiently described; attrition is considered for repeated assessments. | L: nutritional outcome and major clinical factors are measured with established tools or clearly defined source variables. | M: adjusted model is reported with clinically relevant covariates; residual and unmeasured confounding remain possible. | L/M: model specification and principal estimates are reported; internal validation/diagnostics are available where applicable. | Low-to-moderate concern: outcome ascertainment and traceability are strong; remaining concerns relate mainly to observational design and residual confounding. |
| 20 | Tsai et al. (2020) | Retrospective cohort; PG-SGA prevalence and postoperative complications | M: single-center or convenience/clinical sampling; recruitment is described but representativeness is limited. | M: sample size and core characteristics are reported; some setting, missing-data, or precision details are incomplete. | L: the nutritional instrument and source-defined threshold/time point are explicit and applied consistently. | M: completeness and response handling are not fully detailed; complete-case or small-sample bias is possible. | M: participation is adequately described, although cross-sectional/convenience selection may limit representativeness. | L/M: the principal outcome is measured using a recognized tool; some factor measurement details are limited. | M/H: prevalence-focused or mainly unadjusted analysis; confounding control is limited for association claims. | M: analysis is appropriate to the design; precision, multiplicity, or small-event reporting is incomplete in places. | Moderate concern: the study is usable for its prespecified contribution, with limitations from single-center/small-sample design, incomplete adjustment, or response reporting. |

***Note.*** *JBI prevalence domains were applied to sampling, sample description, outcome measurement, and completeness/analysis. QUIPS domains were applied to participation/attrition, factor and outcome measurement, confounding, and statistical analysis/reporting for studies contributing associated-factor or prognostic evidence. For prevalence-only studies, QUIPS judgments describe the degree to which association claims would be supported and do not override the JBI prevalence assessment. L, low concern; M, moderate concern; H, high concern. Overall judgments are aligned with the quality score and level reported in Table 1 while retaining domain-specific reasons.*

**Supplementary Table S4. Full-text Screening Decisions and Eligibility Assessment Records**

| **Decision** | **Category** | **Reason** |
| --- | --- | --- |
| Included | Eligible HNC cohort | Extractable malnutrition prevalence or nutritional-risk estimate. |
| Included | Associated-factor analysis | Extractable adjusted effect estimates. |
| Excluded | Population | Non-HNC population or mixed cancer population without HNC-specific data. |
| Excluded | Outcome | No nutritional assessment or malnutrition outcome. |
| Excluded | Data | No extractable prevalence or associated-factor estimate. |
| Excluded | Publication type | Review, editorial, protocol, or abstract only. |
| Excluded | Overlap | Duplicate or overlapping cohort. |

**Supplementary Table S5. Number of Excluded Full-text Articles and Reasons for Exclusion**

| **Exclusion reason** | **Number of excluded reports** | **Description** |
| --- | --- | --- |
| No malnutrition outcome | 83 | No nutritional assessment or malnutrition endpoint. |
| Non-HNC/mixed population | 64 | No extractable HNC-specific subgroup. |
| No extractable data | 44 | No prevalence or associated-factor estimate. |
| Non-original publication | 39 | Review, editorial, protocol, or conference abstract. |
| Duplicate/overlapping cohort | 18 | Overlapping publication identified during full-text review. |
| Unavailable/other reasons | 29 | Language, definition, or population-related exclusion. |

**Supplementary Table S6. Effect-size Calculation Procedures**

| **Effect estimate** | **Calculation** | **Purpose** |
| --- | --- | --- |
| Prevalence | p = events / total; logit transformation used. | Pooling prevalence using REML random-effects model. |
| OR transformation | $log\left( OR \right)$; $SE=$[$log\left( upperCI \right)-log\left( lowerCI \right)$]/3.92. | Pooling associated factors. |
| Heterogeneity | tau² estimated by REML; I² from Q statistics. | Quantifying between-study variability. |
| Multiple testing | Benjamini-Hochberg adjustment. | Generating q-values. |

**Supplementary Table S7. Sensitivity-analysis Results**

| **Analysis** | **Method** | **Conclusion** |
| --- | --- | --- |
| Leave-one-out analysis | Sequential removal of individual studies. | No single study changed the overall interpretation. |
| Alternative model | GLMM sensitivity analysis. | Results remained consistent with REML estimates. |
| Alternative transformation | Alternative variance transformation. | Conclusions remained stable. |
| Tool-specific analysis | GLIM, PG-SGA, NRS-2002 subgroup analyses. | Assessment-specific differences were preserved. |
